# Machine learning, whole genome sequencing, and Mendelian randomization support a role of CRP on COVID-19 severity

**DOI:** 10.1186/s10020-026-01512-6

**Published:** 2026-05-27

**Authors:** Francesca Lantieri, Stefania Croci, Sergio Decherchi, Marta Rusmini, Giada Recchi, Martina Bonacini, Ilaria Ferrigno, Alessandro Rossi, Yeraldin Chiquinquira Castillo De Spelorzi, Edoardo Henzen, Francesca Rosamilia, Davide Cangelosi, Fabio Landuzzi, Andrea Angius, Vincenzo Rallo, Pamela Mancuso, Annamaria Pezzarossi, Paolo Giorgi Rossi, Mariagrazia Catanoso, Marco Gattorno, Andrea Cavalli, Pier Luigi Meroni, Diego Vozzi, Paolo Uva, Isabella Ceccherini, Carlo Salvarani

**Affiliations:** 1 Biostatistics Unit, Department of Health Sciences (DISSAL), University of Genoa, Genoa, Italy; 2 Clinical Bioinformatics Unit, IRCCS Istituto Giannina Gaslini, Genoa, Italy; 3 Unit of Clinical Immunology, Allergy and Advanced Biotechnologies, Azienda USL-IRCCS di Reggio Emilia, Reggio Emilia, Italy; 4 Fondazione Istituto Italiano di Tecnologia, Genoa, Italy; 5 Rheumatology and Autoinflammatory Diseases Unit, IRCCS Istituto Giannina Gaslini, Genoa, Italy; 6 Genomics Facility, Fondazione Istituto Italiano di Tecnologia, Genoa, Italy; 7 Computational Genomics, Center for Clinical and Computational Genomics (C3G), Italian Institute of Technology, Aosta, 11100 Italy; 8 Institute of Genetics and Biomedical Research, National Research Council, University Campus of Cagliari, Monserrato, Cagliari, Italy; 9 Unit of Epidemiology, Azienda USL-IRCCS di Reggio Emilia, Reggio Emilia, Italy; 10 Unit of Rheumatology, Azienda USL-IRCCS di Reggio Emilia, Reggio Emilia, Italy; 11 Computational and Chemical Biology, Fondazione Istituto Italiano di Tecnologia, Genoa, Italy; 12 European Center for Atomic and Molecular Computing, Swiss Federal Institute of Technology, Lausanne, Lausanne, 1015 Switzerland; 13 Laboratory of Immunorheumatologic Researches, IRCCS Istituto Auxologico Italiano, Milan, Italy; 14 UOSD Research Laboratories Aggregation Area, IRCCS Istituto Giannina Gaslini, Via Gerolamo Gaslini, Genoa, 5 - 16147 Italy; 15 University of Modena and Reggio Emilia, Modena, Italy

**Keywords:** Interpretable Machine Learning, Mendelian Randomization, C-Reactive Protein, CRP causal role, COVID-19 severity prediction, TFEB

## Abstract

**Background:**

The coronavirus disease 2019 (COVID-19) ranges from asymptomatic to very severe infection and death, largely depending on host factors, including genetics. We have investigated clinical and genetic data from 200 COVID-19 patients to search for factors predisposing to increased disease severity.

**Methods:**

Patients were divided into non-hospitalized mild/pauci-symptomatic and hospitalized severe. An interpretable Machine Learning approach was applied to blood biomarkers while genome-wide associations were performed for COVID-19 severity. Finally, a possible causal role of chronic low-grade inflammation on COVID-19 severity was searched by Mendelian Randomization.

**Results:**

A high severity predictive role was observed in our sample by Machine Learning for the C-Reactive Protein measured in the course of SARS-CoV-2 infection (iCRP). This was also suggested by evidence of association with variants known to be involved in the CRP levels in the general population (pCRP). Finally, a possible causal role of chronic low-grade inflammation on COVID-19 severity could be shown by Mendelian Randomization using publicly available summary statistics of two COVID-19 Genome-Wide Association Studies.

**Conclusions:**

Consistent with previous results, a predictive role of CRP levels on COVID-19 severity was detected in our sample. Furthermore, Mendelian Randomization supported a causal role of genetically predicted chronic CRP levels.

**Supplementary Information:**

The online version contains supplementary material available at 10.1186/s10020-026-01512-6.

## Background

Infections with the Severe Acute Respiratory Syndrome (SARS) associated coronavirus 2 (SARS-CoV-2) can lead to the coronavirus disease 2019 (COVID-19), characterized by a broad spectrum of clinical manifestations, ranging from asymptomatic infections to tissue damage, organ failure and death (Carvajal et al. 2024). While the majority of patients have a mild disease, up to 20% develop a severe interstitial pneumonia with high levels of acute phase mediators, uncontrolled inflammatory response with massive release of cytokines (cytokine storm), diffuse lung edema, inflammatory cell infiltration and disseminated coagulation (Guan et al. 2020; Henderson et al. 2020). Such a clinical picture has many features of acute respiratory distress syndrome (ARDS) and is the main cause of morbidity and mortality in patients with COVID-19 (Guan et al. 2020; Henderson et al. 2020).

Understanding the role of individual genetic variation in conferring differential susceptibility to viral infections and disease severity, as well as in determining differential responses to treatment, still requires improvement. Furthermore, sufficient biomarkers capable of predicting differential disease progression are not yet known (Cappadona et al. 2023; Bastani and Jalilian 2024; Ebrahimi et al. 2024; Singh et al. 2024; Yip et al. 2024; Dubrovskyi et al. 2025). For this purpose, different approaches can be adopted.

Extensive studies, carried out to investigate genetic predisposition to COVID-19, have identified dozens of susceptibility loci variously overlapping between three main phenotypes (COVID-19 Host Genetics Initiative 2021, 2023; Roberts et al. 2022): (i) hospitalized vs. general population or vs. not hospitalized; (ii) severe vs. not severe COVID-19, and (iii) SARS-CoV-2-positive vs. SARS-CoV-2-negative individuals (https://www.covid19hg.org/). Overall, the heterogenous phenotype of COVID-19 is determined by a complex interplay between non-genetic factors (e.g. age, sex, socioeconomic features) and genetic variants with small effect (Zsichla and Müller 2023). Genetic studies have so far been conducted in large sample sizes and through meta-analysis to detect statistically significant genetic associations (Ellinghaus et al. 2020; Shelton et al. 2021; COVID-19 Host Genetics Initiative 2023; Pairo-Castineira et al. 2023).

In COVID-19, Machine Learning (ML) techniques have also been employed to identify cardiovascular biomarkers as prognostic of in-hospital mortality in COVID-19 patients (Gustafson et al. 2022) and tissue-infiltrating immune cells, age, cognitive status, complications, and inflammatory biomarkers as classifiers (Li et al. 2024a; Rajwa et al. 2024). However, published models often lack interpretability and transferability (Roberts et al. 2021), and biases in the data need to be addressed (Bassi et al. 2024). Finally, several factors have been investigated for their causative effect on COVID-19 severity by Mendelian Randomization (MR), such as diet (Li et al. 2024b), circulating inflammatory proteins (Baranova et al. 2024), and childhood Body Mass Index (BMI) (Liu et al. 2024).

In the present study, we have investigated COVID-19 patients’ clinical data by an interpretable ML approach and whole genome sequencing data by genetic association, in addition to applying a MR analysis on publicly available data. Triangulation of the evidence provided by these approaches confirms the elevated CRP levels in severe COVID-19 (iCRP, during infection) and suggests the causal role of CRP genetic predisposition to high chronic CRP levels (the CRP levels in the general population, before COVID-19 infection, pCRP).

## Methods

### Patients’ characteristics and inclusion criteria

**Fig. 1:**
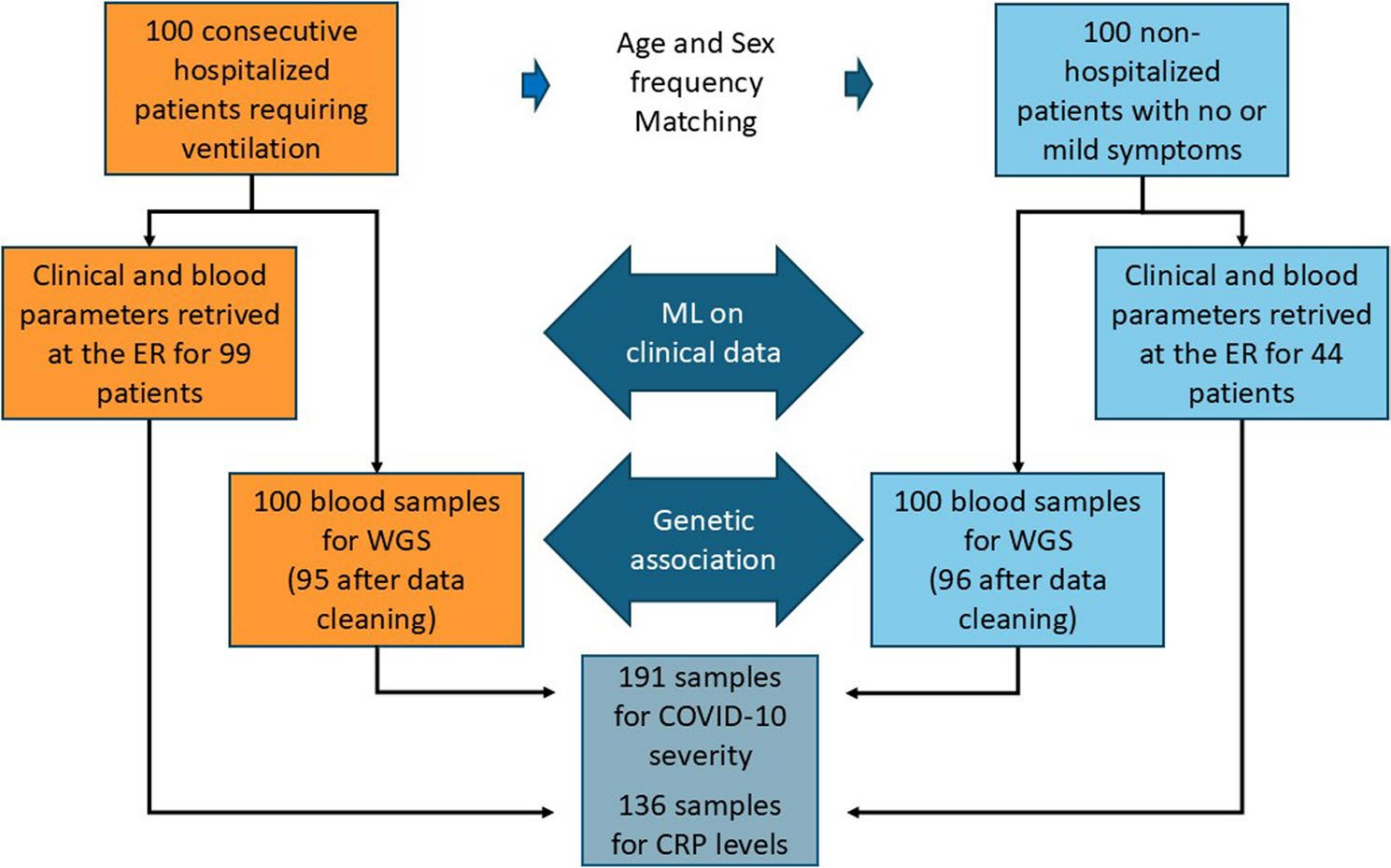
Flowchart of patients’ enrollment and analysis. One hundred hospitalized patients consecutively diagnosed with SARS-CoV-2 infection, fulfilling the inclusion criteria, and belonging to phenotypes 4 and 5 (Galli et al. 2022) constituted the hospitalized/severe group. A hundred patients who did not require hospitalization and belonging to phenotypes 1 and 2 A (Galli et al. 2022) constituted the mild group and were retrospectively selected from consecutive eligible patients to include patients with age and sex distribution similar to that of the severe group. Clinical data and blood sample for genetic investigation were collected for all of them at the emergency room (ER) or, in the case of mild subjects that had not been visited at the ER, at a second agreed visit after the end of quarantine. These 200 patients are shown at the top of the figure. The blood sample aimed at the biomarkers dosage measurements were collected at the emergency room (ER) visit, performed for 99 of the severe and 44 of the mild patients. ML on blood biomarkers was performed on these 143 patients. The 200 blood samples for the genetic analysis underwent DNA Whole Genome Sequencing (WGS). After WGS data cleaning, 191 patients (95 with severe and 96 with mild COVID-19) were used to perform COVID-19 severity genetic association, 136 of which were also used to perform the genetic association with the measured CRP levels

To identify genetic variants associated with COVID-19 severity, we enrolled two groups of patients, one with severe disease and one with mild symptoms (Fig. 1). The first group included 100 hospitalized patients consecutively diagnosed with SARS-CoV-2 infection from March 2020 to May 2020 and having received non-invasive ventilation or intubated during the hospitalization. They belonged to phenotypes 4 and 5 based on Galli et al. (Galli et al. 2022). We excluded patients with other infections. A hundred subjects who did not require hospitalization and belonged to phenotypes 1 and 2 A (Galli et al. 2022) were age and sex frequency matched to the severe group, retrospectively selected from the lists of cases reported to Public health service. These mild patients could have rhinitis for maximum 3 days, fever < 37.5 °C for 1 day, astenia for 1 day, anosmia or aguesia. Only 44 of them had an emergency room visit in the same period as the severe group, at which the standard blood tests were performed.

Given the enrollment period, none of the individuals included in the study was COVID-19 vaccinated. Inclusion criteria were: age > 18 years, positive for SARS-CoV-2 by real-time PCR on nasopharyngeal and oropharyngeal swabs, agreeing to participate in the study and willing to give a blood sample for the genetic analysis. The severe group had received non-invasive ventilation or intubated during the hospitalization (Table 1).

**Table 1:** Clinical and laboratory characteristics of the COVID-19 patients

	Complete cohort		Sub-cohorts with biomarker data^a^		
	Mild patients *n* = 100	Severe patients *n* = 100	Mild patients *n* = 44	Severe patients *n* = 99	Reference values^b^
Age^c^	62 (52–71)	64 (57–70)	56 (46–62)	64 (57–70)	
Female/Male	38/62	27/73	20/24	27/72	
Hospitalization	0%	100%	0%	100%	
Non-invasive ventilation and/or intubation	0%	100%	0%	100%	
Lung involvement < 20%^d^	100%	0%	100%	0%	
Lung involvement ≥ 20%^d^	0%	100%	0%	100%	
SO2 (%)			98 (97–99)	93 (91–95)	> 94
Days from symptoms onset to blood biomarkers measurement^e^			8.6 ± 7.8	7.6 ± 4.2	
Neutrophils (x1000/mmc)^c^			2.91 (2.57–5.04)	4.53 (3.21–6.65)	1.60–7.50
Lymphocytes (x1000/mmc)^c^			1.42 (1.01–1.83)	0.82 (0.69–1.04)	0.80-4.00
Monocytes (x1000/mmc)^c^			0.44 (0.33–0.58)	0.27 (0.22–0.41)	0.08-1.00
Platelets (x1000/µL)^c^			202 (171–260)	182 (152–254)	150–450
LDH (U/L)^c^			386 (299–453)	615 (513–813)	208–378
CRP (mg/dL)^c^			0.56 (0.10–1.33)	11.29 (5.50-16.83)	0.00-0.50
Comorbidities	*n* = 95	*n* = 96	*n* = 40	*n* = 96	
≥ 1 comorbidity	16 (17%)	23 (24%)	5 (13%)	23 (24%)	
≥ 3 comorbidities	3	2	0	2	
Rheumatologic disease	1	0	0	0	
Cancer	8	7	0	7	
Diabetes without complications	3 (3.7%)	13 (13.5%)	0	13 (13.5%)	

*Abbreviations: SO2* oxygen saturation, *LDH* Lactate Dehydrogenase, *CRP* C-reactive protein

^a^Data were measured maximum 7 days after the emergency department presentation. Blood markers, SO2, and lung involvement data were available for 44 mild patients and 99 severe patients

^b^Reference intervals of laboratory reports from our institution

^c^Median values with interquartile ranges are reported

^d^Visual scoring system which considered any pathological changes likely due to COVID-19, on chest X-rays and/or high-resolution computed tomography

^e^Mean ± Standard Deviation

**Fig. 2:**
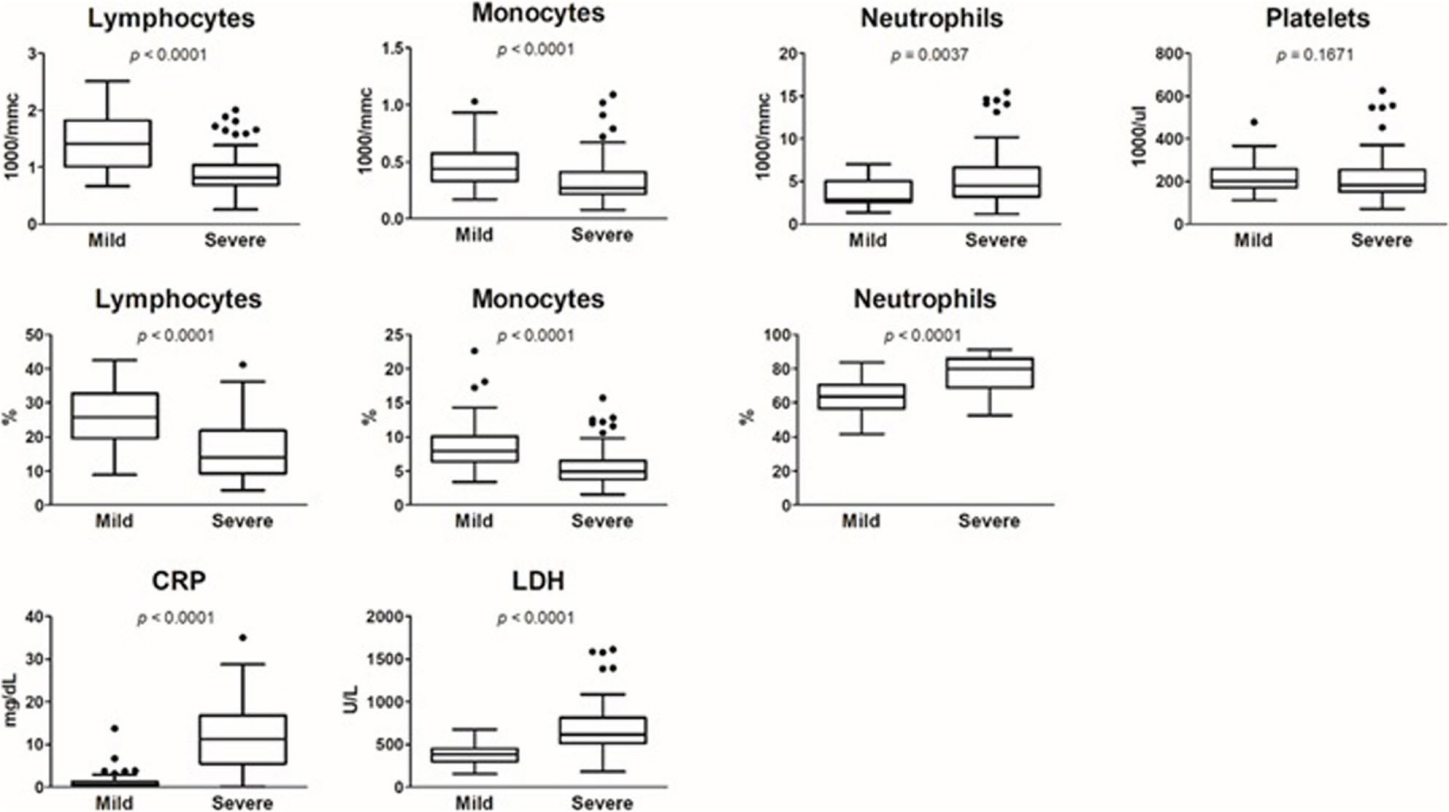
Laboratory data of mild and severe COVID-19 patients. Concentrations (upper panels) and relative percentages (middle panels) of lymphocytes, monocytes and neutrophils are shown as well as concentrations of platelets, C reactive protein (CRP) and lactate dehydrogenase (LDH). Data are shown as box and whiskers. Box edges are the 25th – 75th percentiles with the median lines. Tukey whiskers are shown. Data were collected at maximum 7 days after the emergency department presentation and were analyzed by Mann Whitney test

All data, including symptoms, diagnosis, hospitalization, and laboratory and imaging results were retrieved from hospital electronic records, the COVID-19 Surveillance Registry, Reggio Emilia Department of Public Health’s epidemiological investigations, contact tracing and symptom surveillance for people in self-isolation. C reactive protein (CRP) and lactate dehydrogenase (LDH) levels, lymphocyte, neutrophil, monocyte, and platelet counts, and arterial blood gas analysis data were measured as standard blood test at the emergency room visit, or, when, in case of hospitalized patients, the measurement at emergency room was not present, we considered the closest blood samples to admission, in any case, no more than 7 days after the emergency department presentation (Fig. 2). The blood sample for the genetic analysis was collected at the emergency room, or, in the case of mild subjects that had not been visited at the emergency room, at a second agreed visit after the end of quarantine. Further details on subject recruitment and clinical phenotypes are reported in Supplementary Text.

The study was approved by the Area Vasta Emilia Nord (AVEN) Ethics Committee on 20th October 2020 (protocol number: 1051/2020/OSS/AUSLRE – WGS_COVID-19). All participants signed the informed consent form.

### DNA extraction, genome sequencing, and data cleaning

DNA was extracted from peripheral blood and underwent Whole Genome Sequencing (WGS). Briefly, 200 ng of genomic DNA from each individual were used to construct barcoded genomic libraries that, once pooled, were sequenced using the Illumina NovaSeq 6000 system (Illumina, Inc.) (Supplementary Text). The FastQ data thus obtained were processed through the commercial Illumina DRAGEN (Dynamic Read Analysis for GENomics) Bio-IT Platform, based on GATK Best Practices, by applying the Germline Pipeline (v.3.10.4) on all single samples using the genome assembly GRCh38 (hg38) as reference. The overall mean coverage was 30.1X with an overall percentage of sites with at least 20X coverage of 88.4%. The mean duplicate rate was 13.6% (median = 14.6%). VCF files were annotated for SNVs and indels variants, also including allele frequencies, pathogenicity and splicing predictions, conservation scores and the ClinVar classification.

The detected variants were filtered out if not passing the standard sequencing quality criteria, with high missingness (> 5%), and deviating from Hardy-Weinberg Equilibrium (*p* < 10 − 6). Samples with different ancestry, sex discordance, relatedness, > 3% genotype missingness, or abnormal heterozygosity, were further removed. Following data cleaning, nine patients were excluded (191 final patients’ cohort) and 6.389.143 variants located on autosomal chromosomes resulted available for genetic analyses (see Supplementary Text, as well as Supplementary Fig. S1 and S2 for details).

### Machine learning analysis

A machine learning analysis was performed on clinical data to discriminate the mild from the severe status via an interpretable classification model. To this end, among other interpretable machine learning models, we took advantage of a Decision Tree Classifier as capable of discovering a set of human interpretable “if-then-else” rules to classify a sample, select appropriate features, and define explicit decision threshold values (Supplementary Text).

This model was chosen because of its interpretability of the learnt rules (easy to apply in clinical routine) and easy of overfitting control (tree depth) hence rendering the approach overall trustworthy. The Supplementary Text discusses additional results obtained from the logistic regression model and from the RandomForest method used for probability calibration; both models confirm the Decision Tree findings and are still interpretable (yet without decision thresholds). We included in the model only the quantitative and blood-derived quantities (leukocytes, neutrophils, lymphocytes, and monocytes count and percentages, platelets count, and LDH and CRP levels). At the technical level we used a tree of type CART (Breiman et al. 1984) implemented in the Scikit-learn library version 1.2.2 (Pedregosa et al. 2011) in Python language. We used random shuffling and repeated stratification to perform in-cohort validation, together with max depth of 2.0 levels (4 leaves) hence 3 decision variables at most to prevent overfitting. Threshold uncertainty confidence intervals have been estimated via the Chebyshev (distribution-type-independent) inequality at the 95% probability. Classification probability for new data is estimated via the associated leaf purity, namely the ratio of samples of the majority class. To evaluate the model, we used as metrics: accuracy, balanced accuracy, precision, recall, AUC, PR-AUC, and Brier Score, and confusion matrices. All these scores taken together allow us to assess in this cohort the presence of overfitting and the quality of probability estimates. Python 3.10 was also used to pre-process the data and feed the algorithm. The script used to run the analysis is available at the repository https://gitlab.iit.it/SDecherchi/foreum.

### Genetic association analysis

Following data cleaning, a genetic association analysis was performed for COVID-19 severity (hospitalized/severe cases vs. not hospitalized/mild cases). A genome-wide association study (GWAS) was carried out at the purpose of checking for the p-values distribution and for the Lambda Genomic control inflation factor λ (based on median chi-squared values divided by the expected median of the chi-squared distribution). We also carried out an association analysis for the variants reported as significantly associated with COVID-19 phenotypes by Host Genetic Initiative (HGI), release #7 (https://app.covid19hg.org/), to check for the robustness of our data. To this aim we focused in detail on 11,341 variants reported as associated with a *p* < 0.0001 and that were present in our dataset after data cleaning. In addition, we analyzed the 12 variants investigated by Roberts et al. (Roberts et al. 2022) as proxy for 8 COVID-19 phenotypes, among which “Hospitalized vs. Not Hospitalized”, “Symptomatic vs. Paucisymptomatic”, and “Severity score”. We also carried out a genetic association on the 266 variants reported as independently associated with the CRP levels in the general population by Said et al. (Said et al. 2022). Finally, we performed a GWAS as well as the association analysis between pCRP candidate variants and iCRP levels for individuals for which the data were available in our sample. Data transformation and the selection of relevant covariates among sex, age, and comorbidities (defined as the presence of any comorbidity among those collected, see Supplementary text for the complete list) to be included in the model were performed with R 4.3.2. (R Core Team 2020). Nevertheless, we repeated the analyses including all the above covariates and the first 10 principal components, estimated by the multidimensional scaling analysis implemented in plink v1.07, as a sensitivity analysis. Genetic association analyses were performed using plink v1.07 by chi-square or linear regression (http://pngu.mgh.harvard.edu/purcell/plink/) (Purcell et al. 2007). Manhattan plot and qqplot were drawn with R 4.3.2. (R Core Team 2020). The probability of obtaining a number of nominally significant variants equal or higher than what we have observed for CRP levels and/or for the severe COVID-19 phenotype was calculated by the binomial distribution. The probability that variants nominally significantly associated with COVID-19 severity were also associated with iCRP by chance was tested by the McNemar test. All tests were 2-tailed, with the level of nominal significance set at alpha = 0.05.

### Mendelian randomization

We applied a two-sample Mendelian Randomization (MR) analysis to COVID-19 as the outcome using the CRP levels as the exposure to investigate the causal effect of the genetically predisposed levels of CRP on COVID-19 severity.

MR relies on the three assumptions that the genetic variants selected as Instrument Variables (IV): (1) are strongly associated with the exposure (CRP levels) (Relevance); (2) are not associated with the outcome (COVID-19) via a confounding pathway (Independence); and (3) do not affect COVID-19 directly, but only via the CRP levels (Exclusion restriction).

The 266 variants reported as associated with the level of CRP by Said et al. (Said et al. 2022) by a GWAS on around 500,000 individuals from UKBiobank were used as exposure-associated genetic variants (instrumental variables, IVs). These variants, none located in the CRP gene (trans-SNPs) were significant at a genome-wide level, with F-statistics ranging from 29.7 to 1534.6, and were thus considered as strongly associated (Relevance). Since these variants are reported as independently associated with the CRP levels by the authors, we did not carry out any LD-based pruning. However, we performed the analysis also after LD pruning at r2 < 0.01 at a window size of 500 kb as a sensitivity analysis (Supplementary Text) to check for bias due to correlation between the IVs. Given that the IVs should act on the outcome only through the exposure, and to check for a possible reverse causal effect of the IVs acting on the exposure through the outcome, we repeated the analysis excluding those variants more associated with the outcomes than with the exposure (Steiger filtering) (Hemani et al. 2017). Additionally, we searched the PheWeb database to check whether the variants might have a confounding effect on the outcome through different risk factors (Independence assumption); to this aim, we excluded those variants associated to endocrine/metabolic disorders such as obesity, diabetes, circulatory system or digestive traits with a *p* < 10^− 5^.

We extracted the summary statistics for these IVs from the HGI GWAS meta-analyses (https://www.covid19hg.org/results/r7/), using both the severity phenotype (A2_ALL_eur_leave_23andme panel: respiratory critically ill COVID-19 patients vs. general population), for which the European cohort was available, and the hospitalization phenotype (B1_ALL_leave_23andme: hospitalized vs. not hospitalized COVID-19 patients). In addition, we selected the study by Ellinghaus et al. (Ellinghaus et al. 2020) from the GWAS Catalog (Sollis et al. 2023), conducted on respiratory failure COVID-19 patients vs. blood donors controls, because it was carried out on Italian and Spanish samples.

We applied the multiplicative random-effect inverse-variance weighted (IVW) method implemented in the Mendelian Randomization package, version 0.10.0 (Yavorska and Burgess 2017), R Software 4.3.2. (https://www.R-project.org/) (R Core Team 2020^2020^) as the primary analysis (Bowden et al. 2016). The weighted median (WM), the simple median (SM), the MR-Egger (Bowden et al. 2015), and the Mendelian Randomization Pleiotropy Residual Sum and Outlier (MRPRESSO R package, version 1.0) (Verbanck et al. 2018) methods were employed as sensitivity analyses to investigate pleiotropy (Exclusion restriction). To assess the robustness of the results and presence of directional pleiotropy and heterogeneity, the Cochran’s Q test and the I^2^ statistics were calculated. Funnel plots, forest plots, and Leave-one-out analysis (Corbin et al. 2016) were also conducted. Finally, we performed a sensitivity analysis with variants within the CRP locus (cis-CRP IVs) (Supplementary text).

To evaluate if there was an effect in the opposite direction, and whether genetical predisposition to severe COVID-19 might have a causal effect on pCRP levels, we carried out a MR using severe COVID-19 as the exposure and the pCRP levels as the outcome. To this end, we selected the variants associated with COVID-19 severity (*p* < 5 10 − 8) from the HGI A2 dataset and the summary statistics by Said et al. (Said et al. 2022), obtaining 3317 variants. Forty-three independent variants were retained after linkage disequilibrium (LD) pruning (r2 < 0.01 at a window size of 500 kb), and were used for the two sample Mendelian Randomization analysis. See Supplementary Text for more details.

## Results

### Enrollment of patients

To identify genetic variants associated with COVID-19 severity we included 200 patients diagnosed with SARS-CoV-2 infection, defined as severe hospitalized (100 patients) and mild pauci-symptomatic non-hospitalized (100 patients) (Galli et al. 2022), with similar age and sex distribution (Fig. 1). Laboratory data could be retrieved from 99 patients in the severe group and 44 subjects in the mild group (Table 1; Fig. 2). These data were taken at the emergency room visit for the mild patients and for 60 of the severe patients, and up to 7 days later for the other 39 severe patients. The time from symptoms onset to blood biomarkers measurements, which can reflect COVID-19 phases, was similar between the groups (7.5 ± 4.2 vs. 8.6 ± 7.8 days).

Severe patients had higher iCRP (11.29 vs. 0.56 mg/dL, *p* < 0.01 by Mann-Whitney test), LDH (615 vs. 386 U/L, *p* < 0.01 by Mann-Whitney test), and neutrophil levels (4.53 vs. 2.91 × 1000/mmc, *p* < 0.01 by Mann-Whitney test), and lower lymphocyte (0.82 vs. 1.42 × 1000/mmc, *p* < 0.01 by Mann-Whitney test) and monocyte levels (0.2 vs. 0.44 × 1000/mmc, *p* < 0.01 by Mann-Whitney test). As expected, patients did not differ regarding age (Student’s t-test *p* = 0.24) and gender (Fisher’s exact test *p* = 0.13).

The presence or absence of comorbidities was retrieved for 95/100 mild patients and 97/100 severe patients. Sixteen out of 95 (17%) mild patients and 23/97 (24%) severe patients had at least one comorbidity. There were no apparent differences in the comorbidities between the two groups, except higher frequency of diabetes without complications in patients with severe COVID-19 (*p* = 0.0164, Fisher’s exact test).

### Interpretable machine learning analysis of the data

We used a decision tree to deliver interpretable “if-then-else” rules to identify the most discriminating factors of disease severity in our sample of 143 individuals (99 severe and 44 mild COVID-19 patients) for which the blood biomarkers measures were available with no missing values. All the patients come from the same source hence there is no external cohort validation. To improve the results reliability in absence of the external validation, we shuffled and split the data into training and validation in the usual 70%-30% proportion (100 + 43 samples) 1000 times, generating 1000 decisions trees. We took advantage of stratification to ensure that the original class proportion is maintained in data split. We computed accuracy, balanced accuracy, precision, recall, AUC, PR-AUC, and Brier Score. Results in Table 2 indicate a robust (low IQR), predictive (high balanced accuracy among others) model for discriminating mild versus severe COVID-19 patients.

**Table 2:** Quality statistics for the decision-tree clinical data classifier

	Accuracy	Balanced Accuracy	Precision	Recall	AUC	PR-AUC	Brier Score
Mean	0.878	0.854	0.915	0.914	0.892	0.926	0.100
Median	0.884	0.859	0.909	0.933	0.900	0.929	0.095
Standard Deviation	0.044	0.059	0.048	0.061	0.060	0.041	0.037
IQR	0.047	0.077	0.080	0.100	0.076	0.055	0.047

*Abbreviations: AUC* Area Under Curve, *PR-AUC* Precision Recall Area Under Curve, *IQR* Interquartile Range

**Fig. 3:**
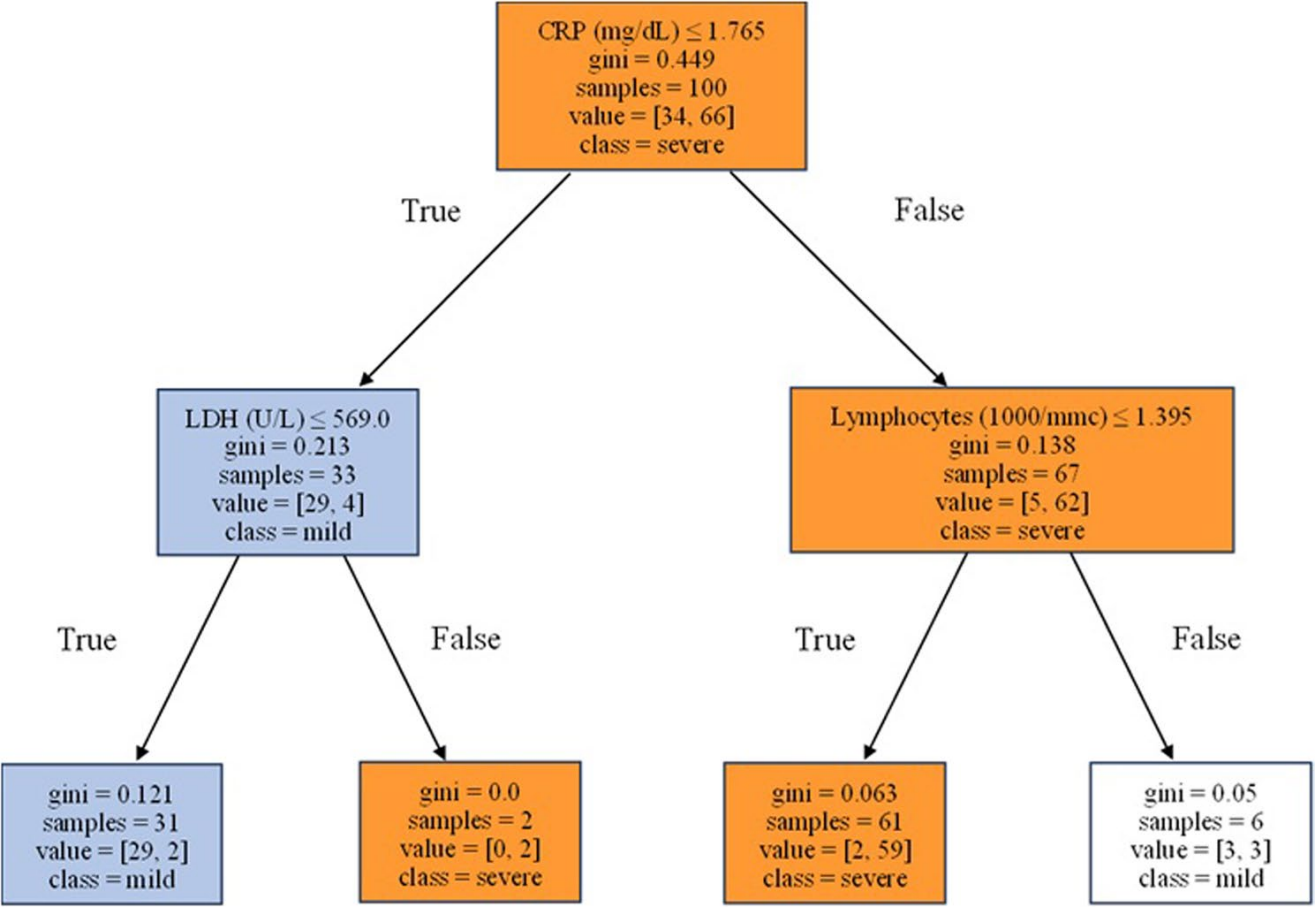
Example decision tree for ‘severe’ vs. ‘mild’ discrimination. The graph shows a decision tree example (out of 100 obtained). The blocks represent the decision variables together with the decision thresholds. Left branches indicate the ‘true’ condition on the decision threshold whereas right indicates ‘false’. In “value” we report the number of samples of the two classes in that leaf. For instance, value=[x, y] indicates that the leaf has x ‘mild’ and y ‘severe’. In “samples” we report the total number of samples in that leaf and the Gini index, where 0 means that the node is all composed by samples belonging to one class and hence is a purity index. The orange, light blue and white colour of the boxes encode respectively the predominance of “severe”, “mild” class or tie

A highly constrained tree (max depth of 2, 3 decision variables) allowed us to get explicit and simple rules for the classification. We detected the features iCRP, LDH, and Lymphocytes, measured in the course of infection, in almost any tree we built (see an example in Fig. 3), with iCRP always scoring first. As we repeated the analysis 1000 times, we were able to obtain an average threshold for the iCRP value (together with its uncertainty, defined as 3 times the standard error) of 2.16 ± 0.08 mg/dL (median 1.76, IQR = 0.075), below which the patient is predicted as ‘mild’ and ‘severe’ otherwise. To confirm this result, we extended the analysis to 5000 repetitions, obtaining the value of 2.18 mg/dL with confidence band = [2.126, 2.242] at 95% probability (via Chebyshev inequality). This threshold proved to be stable as we also tried with 1000 and 2000 splits. We found the distribution of the threshold values is bi-modal yet converged (Supplementary Text). In particular, a lower, more probable, threshold mode around 1.7 mg/dL and a higher, less probable, threshold mode around 4.0 mg/dL recurs across resampling; we also found that in trees where the lower-threshold mode appears as first decision rule, a second CRP split near 4.0 mg/dL frequently appears at the next level. We found that the two different thresholds are not statistically associated with other clinical observables in our dataset but rather are a training result of the learnt trees.

Lastly, we averaged out the confusion matrices over all the repetition using as thresholds 0.2, 0.5, 0.8 in the decision function probability. Table 3 shows the results obtained confirming the modest class overfitting and errors (class 0 is mild, class1 is severe).

**Table 3:** Confusion matrices by changing the probability thresholds

Probability Threshold	Confusion Matrix	
0.2	9.7	3.3
	2.0	27.9
0.5	10.3	2.7
	2.6	27.4
0.8	11.4	1.6
	4.1	25.9

**Fig. 4:**
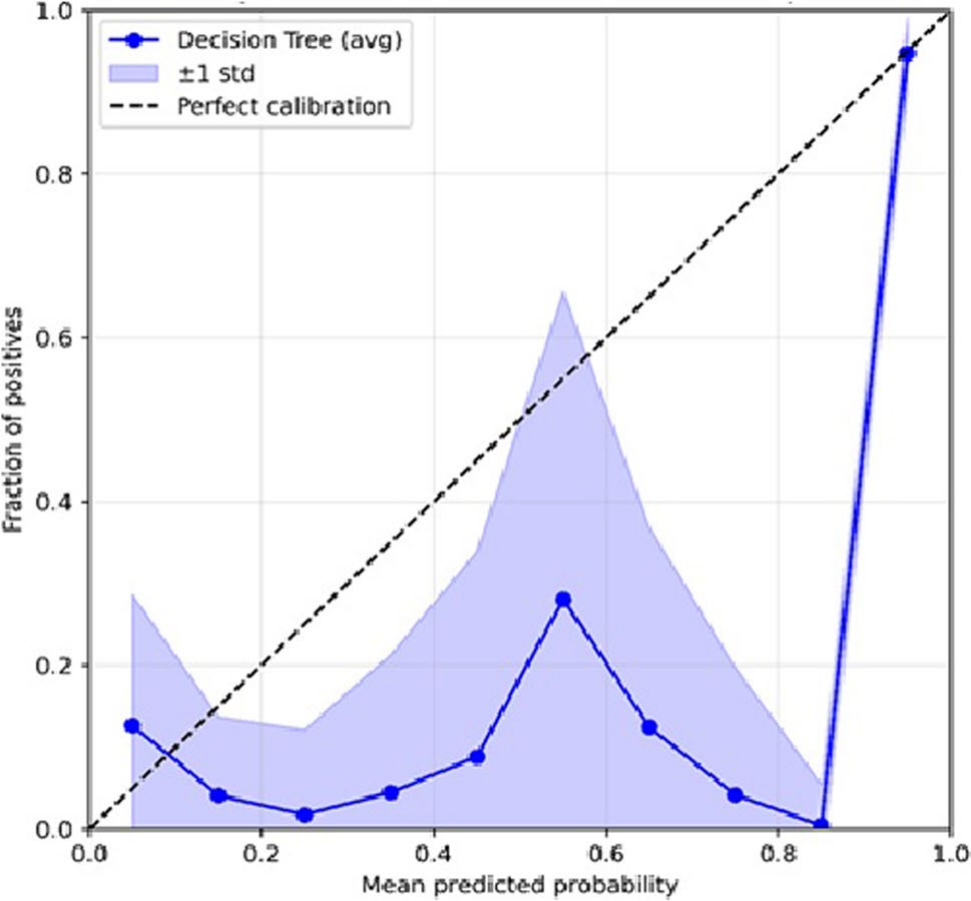
Average calibration curve (Decision tree, depth = 2). Average probability calibration plot for the learnt trees (1000 trials) is shown. The shade region represents one standard deviation. As expected for a tree, which bears discrete probabilities estimates (per leaf), extremes are well calibrated whereas intermediate are much less populated

We also checked the average probability calibration plot in Fig. 4. As expected for a tree, it is not able to capture intermediate probability (it is a very discrete model) yet captures very well the extremes. In the Supplementary Text we provided the calibration curve for averaged Random Forest, which as expected, provides a smoother almost perfect curve, yet threshold-level interpretability in this model is lost. In the Supplementary Text we also provide an analysis based on a still interpretable model, namely logistic regression, which confirms the prominent (first feature selected) role of CRP as correlative factor.

### The genetic profile of the samples confirms the robustness and accuracy of the phenotype ascertainment

In order to obtain a broad genotyping of the 200 samples thus recruited, we opted for the WGS analysis, which would also allow us to obtain information on common and rare variants, not only in the coding region but also at non-coding, genomic level. The sequencing procedure, as well as metrics, sample structure, and data cleaning workflow are reported in the Material and Methods section, in addition to Supplementary Text, Supplementary Table S1, and Supplementary Fig. S1 and S2.

Following data cleaning, nine individuals were excluded because of different ancestry from the rest of the samples, abnormal heterozygosity, and/or genotype call missingness above 3%, with 191 patients remaining for the association analysis. A total of 6.389.143 variants located on autosomal chromosomes, with a MAF ≥ 5% were analyzed for genetic associations. Association with COVID-19 severity was performed comparing hospitalized patients with severe COVID-19 (95 cases) vs. mild, not hospitalized COVID-19 patients (96 patients). Since neither sex and age, as expected by study design, nor complications resulted to be significant covariates on the COVID-19 severity phenotype, we performed genetic association by chi-square test.

The genome-wide analysis supported the good quality of our sample, without apparent population stratification, with a genomic inflation factor lambda (λ) = 1.02 and a QQplot coherent with absence of relevant bias (Supplementary Fig. S3). The association analysis for the COVID-19-associated variants reported by HGI and by Roberts et al. (Roberts et al. 2022) confirmed the consistency of our sample with the hospitalized/severe vs. not hospitalized/mild phenotype (Supplementary Text).

### Genomic loci associated with CRP levels are enriched in the COVID-19 severe samples

Since the machine learning results had strongly indicated iCRP levels as the main clinical factor discriminating mild vs. severe COVID-19 patients, genetic associations on iCRP levels were also carried out. Inverse normal transformed iCRP levels measured for 136 patients at the emergency unit (93 severe and 43 mild patients) and retained after data cleaning were analyzed by linear regression adjusting for sex and age as covariates (Supplementary Fig. S4).

**Fig. 5:**
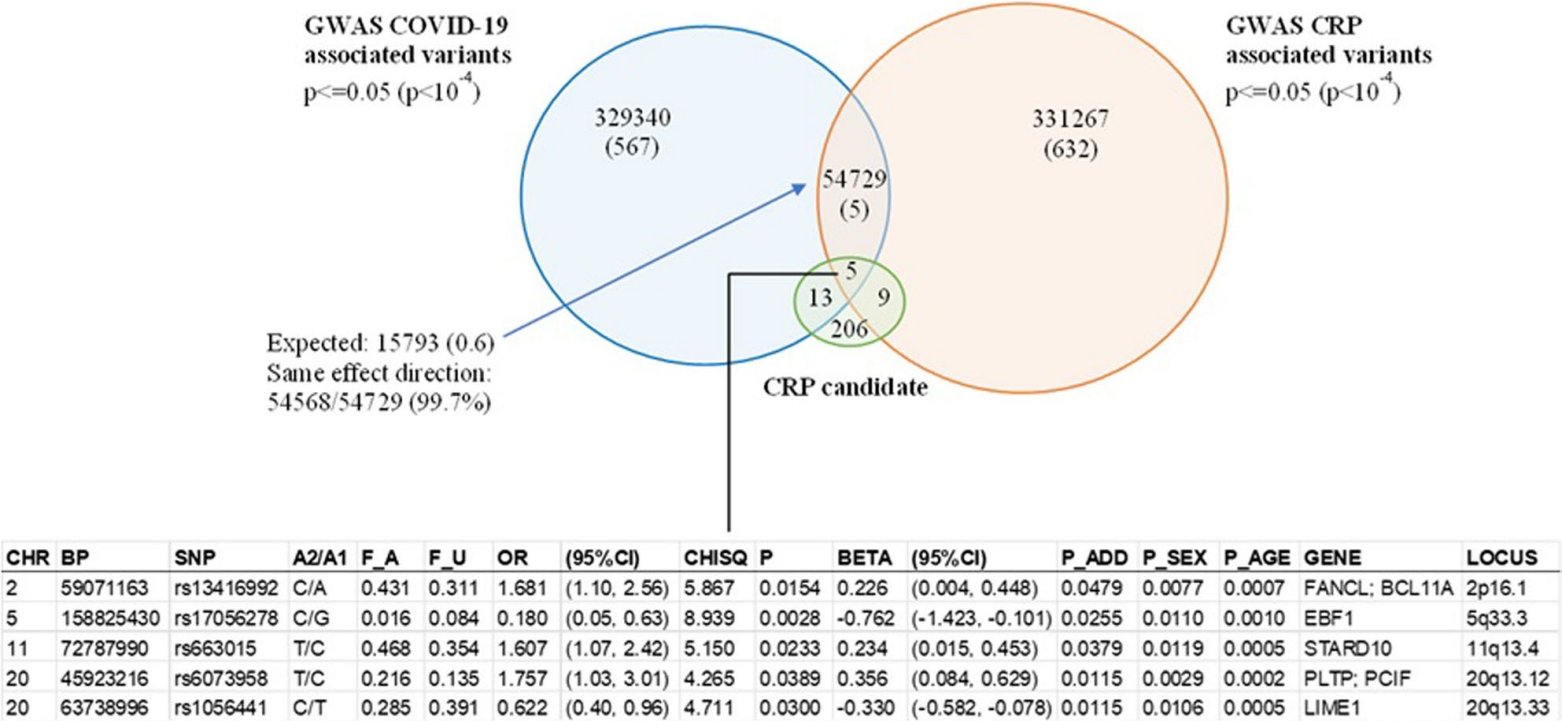
Significant associations overlap between CRP levels and COVID-19 severity in our sample. The Venn Diagram shows that 54,729 variants were nominally significantly associated with both COVID-19 and iCRP (measured after infection occurrence), 99.7% of which with a concordant effect (increased CRP levels and COVID-19 severe cases or decreased CRP levels and mild COVID-19 patients). Considering alpha = 0.05, we would have expected to detect 15,973 (6389143*0.052) variants by chance. Variants with nominal *p* < 0.0001 are reported inside brackets. Of the 223 variants reported to be genome-wide significantly associated with CRP levels by Said et al. (Said et al. 2022) five variants were associated with both phenotypes in our sample, and are reported in the table. A2/A1 are the major and minor allele in our sample; F_A and F_U are the minor allele frequencies in the COVID-19 severe patients and in mild patients, respectively; OR (with 95% confidence interval between brackets) and P are odds-ratio and chi-square p-value for the association with COVID-19 severity; Beta and P_ADD are the beta coefficient and the p-value for the linear regression association with CRP levels; gene is for the gene where the variant is located, two genes separated by a semi-colon are the closest upstream and downstream genes

We first focused on the variants reported as associated with CRP levels in population-based cohorts by Said et al. (Said et al. 2022) under the hypothesis that a low-grade inflammation might predispose to a more severe COVID-19. After data cleaning, 223 of the 266 variants from Said et al. (Said et al. 2022) were also present in our dataset (Supplementary Table S3). Fourteen of these 223 variants were found to be associated with iCRP levels in our sample with a p-value < 0.05, a number slightly higher than the 11 expected by chance. Surprisingly, also for the COVID-19 severity phenotype association there was a higher proportion of nominally significant variants than expected by chance (binomial *p* = 0.0321), with 18 variants nominally significant and one variant surviving Bonferroni correction for multiple tests (rs4714508, the G allele present in 25% of cases and 45.3% of controls, OR [95%CI] = 0.402 [0.260–0.622], *p* = 3.42 × 10-5, in the *TFEB* gene). Five nominally significant variants were associated with both phenotypes, a high number, considering that no variants would have been expected by chance (*p* = 2.7 × 10^− 4^) (Fig. 5). All these variants showed an effect in the same direction for iCRP and COVID-19 severity, with the variants associated with increased CRP levels also associated with the severe form of COVID-19 and viceversa. Interestingly, also considering the variants at a genome-wide level, and not only the above 223 pCRP candidate variants, we found a proportion of nominally significant variants that were associated with both iCRP levels and COVID-19 severity higher than what expected by chance (54729 variants, McNemar’s chi-squared = 6.73, df = 1, *p* = 0.0095), and in almost complete accordance with the effect direction (54568 out of 54729 variants, 99.7%). Variants associated with both phenotypes with *p* < 0.0005 are reported in Supplementary Table S3.

Given that this increased overlap in variants associated to both phenotypes may be due to linkage disequilibrium among those variants, leading to an inflated enrichment, we have repeated the analysis pruning variants in LD (r^2^ > 0.1). The enrichment detected was still evident, with 1632 out of 194,846 associated to both traits (McNemar’s chi-squared = 6.973, df = 1, p-value = 0.008275), 1626 of which with the same effect direction (99.6%), thus highlighting that the observed excess was not due to LD effects (Supplementary text). Finally, our findings were also consistent adding age, sex, comorbidities, and the first 10 principal components as covariates in the association tests (Supplementary Text).

### The causal role of CRP levels in the severity of COVID-19 detected by Mendelian randomization

**Fig. 6:**
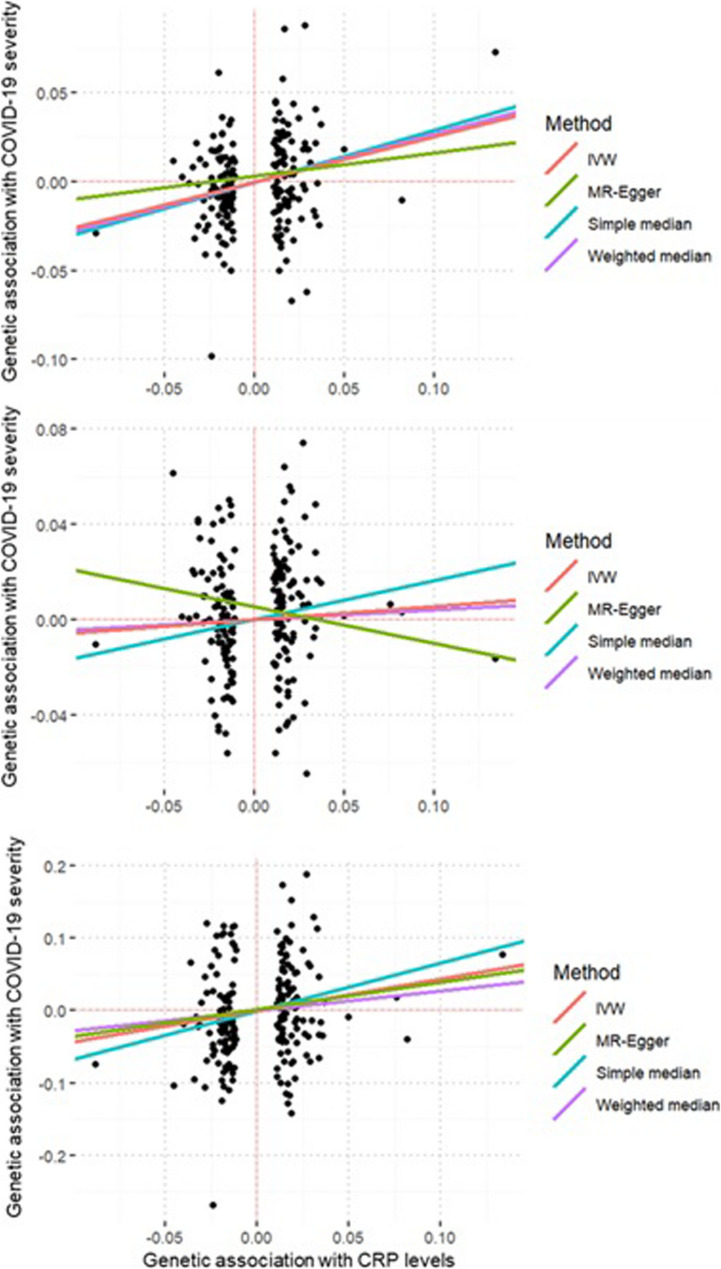
Mendelian Randomization (MR) for three COVID-19 summary statistics GWAS. Scatter plot of genetic associations with the outcome (COVID-19) against genetic associations with the exposure (CRP) are shown. Each dot represents the per allele association of a single genetic variant among those reported as associated with CRP by Said et al. (Said et al. 2022) The causal estimate of CRP levels on COVID-19 are estimated by inverse-variance weighted method (red line), MR-Egger method (green line); simple median method (blue line), and weighted median method (purple line). (A) Across the 204 variants CRP-associated by Said et al. (Said et al. 2022) and present in the summary statistics reported by the HGI meta-analysis on the COVID-19 severe vs. population phenotype (A2_ALL_eur_leave_23andme panel, https://www.covid19hg.org/results/r7/), the genetic increase is correlated with an increase in the odds of severe COVID-19; (B) the graph represents the same analysis performed on the hospitalized vs. not hospitalized phenotype (B1_ALL_leave_23andme). No correlation at all is visible; (C) the genetic correlation with an increased risk of severe COVID-19 severity is visible by MR also on the summary statistics by Ellinghaus et al. (Ellinghaus et al. 2020)

The main analysis to investigate the causal role of the general population CRP (pCRP) on COVID-19 severity suggested that the genetically predicted high pCRP levels predispose to a more severe form of COVID-19 (OR [95%CI]: 1.29 [1.13, 1.48], *p* ≤ 0.0001), but highlighted a high heterogeneity (I^2^ = 45%) (Fig. 6 and Supplementary Table S5). The same analysis performed after Steiger filtering (Hemani et al. 2017) confirmed evidence of a causal effect on COVID-19 severity (OR [95%CI] = 1.14 [1.02, 1.28], *p* = 0.027), with disappeared heterogeneity (I^2^ = 0%) (Supplementary Fig. S6 and Supplementary Table S4 and S5). These analyses were performed by the inverse-variance weighted (IVW) method on COVID-109 severity (A2 panel, critically ill COVID-19 patients vs. general population, from the HGI website) using the pCRP associated variants reported by Said at al. (Said et al. 2022). Of the 266 variants identified by the authors, 250 were present also in the summary statistics of the A2 panel from HGI, and 204 were included in the primary analysis after removing those with ambiguous strand and MAF < 0.05. The F statistic for all variants, estimated through the two-sample MR as an approximation of the first-stage F statistic, was 101.0 (Supplementary Table S5). The F-statistics for each of the 204 single IVs used were above 10, with a mean F of 97.8 (median = 50.5), and a total proportion of variance explained of 4.1% (Supplementary Table S3).

Concordant findings were obtained on the dataset by Ellinghauss et al. (Ellinghaus et al. 2020) (OR [95%CI]: 1.55 [1.04, 2.30], *p* = 0.030), which compared respiratory failure COVID-19 patients vs. blood donors’ controls in Spanish and Italian individuals. No causal effect was detected for the B1_all dataset from HGI phenotype (hospitalized vs. not hospitalized COVID-19 patients).

Results were consistent in sensitivity analyses, with concordant direction and similar size of effect, although statistical significance was not reached with the MR-Egger method. No significant horizontal pleiotropy was detected by this latter method (Supplementary Table S4). Repeating the analysis on the 199 SNPs remaining after LD pruning gave results almost identical to those obtained on the primary analysis on the full set of 204 SNPs (Supplementary Text).

MR-PRESSO global test performed on the 204 IVs before Steiger filtering indicated the presence of horizontal pleiotropy (global test p-value < 10 − 4), with eight variants identified as outliers (three at a significant level) (Supplementary Tables S4 and S6). However, MR-PRESSO results remained significant after correcting for these outliers (Supplementary Table S4). As reported above, high heterogeneity was detected (Cochran’s Q *p* = 0.0000 and I^2^ = 41.5%). Of note, after the removal of the eight outlier variants, the heterogeneity dropped to 14% (Cochran’s Q *p* = 0.0592). Such heterogeneity was detected at a lower extent in the summary statistics by Ellinghauss et al. (Ellinghaus et al. 2020).

Of note, IVW after Steiger filtering (Hemani et al. 2017) supported a causal effect on COVID-19 severity, detected also by the weighted median (*p* = 0.016) and MR-PRESSO (*p* = 0.0009) methods, yet without identifying any heterogeneity or outlier variants (Supplementary Table S4). Interestingly, the 78 variants filtered out with the Steiger method included all the eight outlier variants detected by the MR-PRESSO original analysis (Supplementary Table S6). To check for possible confounding effects on the outcome, we also repeated the analysis by removing those variants found as associated at *p* < 10^− 5^ with any phenotype such as endocrine/metabolic (obesity, diabetes), circulatory system (hypertension), respiratory, etc., as found in the Pheweb database (Supplementary Table S4 and S5), finding generally consistent results.

Despite the heterogeneity among individual variant effect estimates shown by the forest plot, the relatively symmetrical funnel plot and the leave-one-out analyses suggested that no specific variant/s drove the effect, thus supporting the reliability of the IVW main results on all the 204 variants analyzed (Supplementary Fig. S5). The analysis carried out on 5 LD-pruned cis-SNPs showed no significant evidence of causal effect, without any heterogeneity. The effect analyzing together trans- and cis-IV was still visible, but at a reduced extent (Supplementary Table S4).

In the reverse direction based on 43 independent genetic variants, instead, we found a very high heterogeneity (I^2^ = 85.6%, *p* < 0.0001), with a few variants detected as outliers by MR-PRESSO, and no evidence at all of a causal effect of genetic predisposition to severe COVID-19 on the pCRP levels (*p* = 0.864) (Supplementary Fig. S7 and Supplementary Table S4). Similar lack of evidence of causal effect was visible even after adjusting for the outliers by the MR-PRESSO method (Supplementary Table S4).

## Discussion

In this study we have investigated clinical and whole genome sequencing data from COVID-19 patients, comparing severe hospitalized patients requiring ventilation, to mild patients with minimal lung involvement who did not require hospitalization.

The decision trees obtained by a Machine Learning (ML) approach indicated a discriminatory value of the C-Reactive Protein in the course of SARS-CoV-2 infection (iCRP). Higher iCRP levels in severe than in mild COVID-19 patients have already been reported (Stringer et al. 2021). In addition, iCRP has been reported to be a main predictor of COVID-19 severity risk (Sagar et al. 2024) and response to tocilizumab therapy (Cassone et al. 2020). iCRP was also found to discriminate between deceased and survived COVID-19 patients (Yan et al. 2020; Besutti et al. 2022). Our study confirmed higher levels of iCRP in severe than in mild COVID-19 patients. Our ML approach proved, differently from several published ML protocols on this topic, high interpretability and potential transferability. It suggested that the iCRP levels are strongly predictive of COVID-19 hospitalization/severity in our sample, with a discriminatory value of approximately 2.18 mg/dL with very high classification metrics. Transferability is limited by our single current cohort study, which may hide biases, yet our global iCRP threshold between mild/severe classes is consistent with the value of 4.12 mg/dL reported by Yan et al. (Yan et al. 2020), which focused on survival vs. death; this is particularly consistent with our second less probable threshold (bi-modality) found at around 4.00 mg/dL. These findings could be, in perspective, combined obtaining a more accurate semi-quantitative rule: if CRP < 2.18 mg/dL then mild COVID19 and non-increased mortality, if 2.18 < = CRP < 4.12 mg/dL then severe but non-increased mortality and, lastly, if CRP > = 4.12 mg/dL then severe disease and 50% death probability.

CRP levels rise quickly in response to not only infection but also tissue injury and inflammation, typical of clinical deterioration, organ damage and flare-up. High CRP levels are also characteristic of systemic inflammation as well as chronic conditions such as rheumatoid arthritis, cardiovascular diseases (CVD), metabolic syndrome, obesity, atherosclerosis, and diabetes (Momiyama et al. 2010; Shankar and Li 2008; Wee et al. 2008), and several diseases can develop from chronic inflammation (Libby 2002). In our study iCRP levels were measured generally at the emergency room visit, still hours or days after the onset of the infection, thus it is difficult to discriminate whether the increased iCRP levels are an obvious result of the inflammatory process or could be considered as a predictive factor. Also the study by Yan et al. (Yan et al. 2020) reported the discriminatory potential of CRP, however, it was not reported at which time interval from the infection occurrence the CRP levels were measured.

CRP levels in the general population (pCRP) are genetically predicted, with hundreds of loci genome-wide significantly associated with pCRP levels (Said et al. 2022). Interestingly, the pCRP levels were also significantly associated with several respiratory diseases with a causal role, as marker of chronic low-grade inflammation (Stringer et al. 2021). We can therefore postulate that risk factors for baseline level of inflammation could also be risk factors for disease severity in individuals infected with SARS-CoV-2. Consistently, several pre-existing conditions associated with COVID-19 severity are correlated to chronic inflammation (Fritsche et al. 2023). Among these comorbidities, there are respiratory traits (such as airway obstruction), obesity, inflammatory bowel disease, diverticulosis and diverticulitis, hypertension, endocrine/metabolic disorders, diabetes, and alcohol and tobacco-related disorders. Obesity and related metabolic imbalance are known to be associated with chronic low-level inflammation (Hildebrandt et al. 2023).

The sequencing of the whole genome (WGS) has allowed us to investigate variants nominally associated with both the COVID-19 severity and the iCRP levels in our sample, detecting an overlap in the nominally significant genetic association between the two phenotypes. In addition, WGS has allowed us to evaluate the variants already reported by others as associated with CRP (Said et al. 2022). Because the present cohort is clearly underpowered for GWAS studies, none of the COVID-19-associated variants by HGI was significant in our sample after correcting for multiple testing. However, when focusing on specific genomic regions this problem is reduced, as the number of tests that need to be performed decreases. We were thus able to find that several of the known pCRP-associated variants were also associated with COVID-19 severity in our sample, among which a variant still significant after correction for multiple tests localized in the *TFEB* gene, a finding interesting but to be taken cautiously given the small sample size of our study. This gene, involved in several processes, including lysosome localization and positive regulation of autophagy, is particularly attractive since its implication in SARS-Cov-2 has already been suggested. This is the case of the activation of the NLRP3 inflammasome and CASP1, followed by IL1B and IL18 maturation and secretion, as induced by impairment of the autophagic flux (Sun et al. 2022). In addition, the mTOR-mediated phosphorylation of the transcription factor TFEB is necessary for facilitating its nuclear translocation and triggering microautophagy. In this respect, infection of cultured lung epithelial cells with live SARS-CoV-2 resulted in impairment of autophagic flux, activation of NLRP3 inflammasomes, and pyroptosis (Shi et al. 2022). On the other hand, we cannot exclude that among the patients who have experienced more severe COVID-19 inborn errors of immunity might be present, due to rare variants in one of the many genes involved in the complex regulation of the immune system, possibly related to CRP levels.

According to the results we obtained by ML and genetic association analyses, the MR analysis on well powered big size GWAS summary statistics available in literature supports that the genetically predicted levels of pCRP, measured in population-based cohorts, might causally predispose to hospitalization/severity. Reverse causality, on the other hand, was not detected at all. The great heterogeneity detected in our MR analysis might indicate horizontal pleiotropy, but the effect estimates were robust to the diverse sensitivity analysis we performed. In addition, the heterogeneity completely disappeared with Steiger filtering, which controls that the direction of the effect does not indicate reverse causality (Hemani et al. 2017). Among the variants removed, some were detected as outliers by MR-PRESSO. In particular, one outlier variant was in the ABO locus, already well known to be implicated in COVID-19. The heterogeneity was less marked in the study carried out only on Italian and Spanish populations (Ellinghaus et al. 2020), which, in turn showed a strong MR effect considering the low sample size. Thus, we could hypothesize that different ancestries may explain some of the heterogeneity and that severity of COVID-19 may be related to ancestral background.

Although iCRP has previously been studied in COVID-19, finding a significant genetic correlation, no significant results emerged from Mendelian randomization (COVID-19 Host Genetics Initiative 2021). This might be due to a different Instrumental Variables (IV) choice, since the variants in common with our study are very limited. Furthermore, we focused only on Europeans, while the study mentioned above analyzed different ancestries without distinction, with a very high heterogeneity detected. Notably, a non-significant causal association of CRP was detected for COVID-19 hospitalized patients vs. general population (Leong et al. 2021); a more recent study on Asian found a significant causal relationship between iCRP and COVID-19 by MR (Chang et al. 2024), however the exact COVID-19 phenotype was not specified.

The choice of the phenotype is crucial, for both the standard GWAS analysis and the MR analysis. The hospitalized/non-hospitalized phenotype, like the one analyzed in our cohort, has already been shown to be associated with specific loci, not completely overlapping with the severity phenotype, and to be also the one least likely to give significant association signals. This is not surprising: although severity is implicit in hospitalization, yet, the hospital admission is intrinsically heterogeneous, since it might depend on several other factors such as the individual age and comorbidity, as well as the local health system. The finding that pCRP may be causally associated with COVID-19 severity rather than to hospitalization is thus coherent. Nonetheless, the phenotype of our cohort, although following the criteria of hospitalization, was also selected including patients at the two opposite extremes of the severity scale (Galli et al. 2022). This can explain the results we obtained, supporting a difference in severity associated with genetically predicted levels of CRP, and in line with the presence of many variants with small effect that contribute to COVID-19 severity. However, strong heterogeneity was detected. Although it was drastically reduced by Steiger filtering, and the MR-Egger method did not reveal significant pleiotropy for the two main analyses, we cannot exclude that it may be due to variants that influence the exposure through different biological mechanisms. Adiposity can be one of these: body fat is known as a primary driver of chronic, low-grade systemic inflammation, producing elevated levels of Interleukin-6 (IL-6) which then acts to release CRP (Zhao et al. 2023).

Our study presents some limitations. First, the very limited sample size did not allow to carry out a genome-wide level association and limits the robust reliability of the association analyses carried out on the CRP candidate variants. We should also keep in mind that our sample was recruited during the first COVID-19 wave, before vaccination. Although this avoided confounding factors, vaccination campaigns and years of exposure to the virus in the general population might have changed the today host–virus interactions and risk factors. In addition, we cannot exclude that the increased genome-wide overlap between variants nominally significantly associated with both phenotypes is inflated by phenotypic dependence between iCRP and COVID-19 severity, thus, this observation should be interpreted as investigative. Another limitation of our work resides in the lack of an external cohort/test able to support our findings at scale. In the lack of external validation, our results should be interpreted as exploratory analyses aimed at generating hypotheses on causal mechanisms and accuracy of the model should not be interpreted as the potential clinical accuracy of the model in practice. Finally, in addition to the possible presence of pleiotropy discussed above, the likely contribution of pCRP to the risk of a more severe form of COVID-19 observed by MR cannot be interpreted as a definitive causal role in all COVID-19 severity phenotypes, also in consideration that cis-IVs did not uphold a causal effect. Despite these limitations, our combined approaches produced consistent results. The integration of clinical biomarker analysis using interpretable machine learning (ML) proved effective as a tool for pre-screening clinical data, useful for further, more detailed genetic analyses, such as WGS and association studies. MR, in turn, indicates a causal role of CRP in increasing the severity of COVID-19. This could also suggest that CRP is able to exert a causal effect in other low-grade inflammatory conditions, a circumstance that deserves attention in light of possible future infectious disease pandemics (Nyberg et al. 2026).

## Conclusions

In the present study, we have investigated COVID-19 patients’ clinical data by an interpretable ML approach identifying the serum C-Reactive Protein (CRP) measured in the course of SARS-CoV-2 infection (iCRP) as a variable with high discriminatory value. Whole genome sequencing data analysis also suggested a relation between iCRP levels and COVID-19 severity. Finally, a MR analysis on publicly available data supported the causative role on COVID-19 severity of genetically predicted high levels of CRP in the general population (pCRP). This observation might explain the increased COVID-19 severity risk observed for persons with conditions characterized by an underlying chronic inflammation such as obesity, diabetes, cancers and even very intense sport activity. The implications of chronic inflammation on healthcare should also be considered in the perspective of possible future pandemic infections.

## Supplementary Information

Supplementary Material 1.

Supplementary Material 2.

Supplementary Material 3.

## Data Availability

The datasets generated and analyzed during the current study are available from the corresponding author on reasonable request. In addition, the list of variants detected and their frequencies among the datasets are available in the open platform ZENODO (https://doi.org/10.5281/zenodo.20342064). The script used to run the ML analysis is available at the repository [https://gitlab.iit.it/SDecherchi/foreum](https:/gitlab.iit.it/SDecherchi/foreum) .

## Abbreviations

ARDS: Acute Respiratory Distress Syndrome
AUC: Area Under Curve
BMI: Body Mass Index
COVID-19: CoronaVirus Disease 2019
CRP: C-Reactive Protein
DRAGEN: Dynamic Read Analysis for GENomics
GWAS: Genome-Wide Association Study
HGI: Host Genetic Initiative
IQR: InterQuartile Range
IVs: Instrumental Variables
IVW: Inverse-Variance Weighted
LD: Linkage Disequilibrium
LDH: Lactate Dehydrogenase
ML: Machine Learning
MR: Mendelian Randomization
PR-AUC: Precision Recall Area Under Curve
SARS-CoV-2: Severe Acute Respiratory Syndrome (SARS) associated CoronaVirus 2
SM: Simple Median
SNV: Single Nucleotide Variant
SO2: Oxygen Saturation
VCF: Virtual Contact File
WM: Weighted Median
