# Supplementary material for: Machine learning, whole genome sequencing, and Mendelian randomization support a role of CRP on COVID-19 severity
Source: Mol Med. 2026 May 27;32:117. doi: 10.1186/s10020-026-01512-6 (PMC13393844; doi:10.1186/s10020-026-01512-6)
Supplement: Supplementary file 1 — Supplementary Material 1. [file 10020_2026_1512_MOESM1_ESM.pptx]

## Slide 1
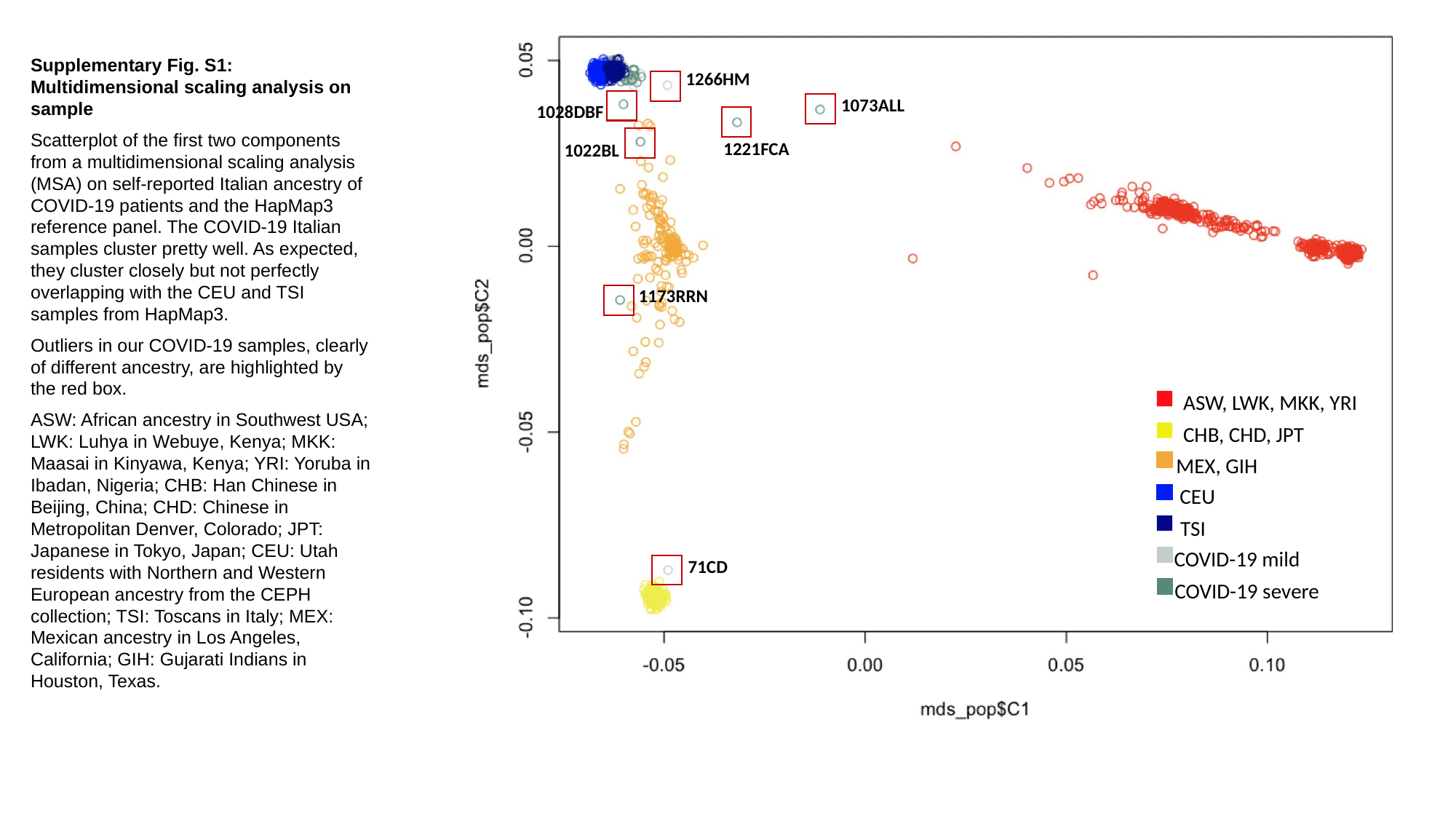

1266HM
1073ALL
1028DBF
1221FCA
1022BL
1173RRN
ASW, LWK, MKK, YRI
CHB, CHD, JPT
MEX, GIH
CEU
TSI
COVID-19 mild
COVID-19 severe
71CD
Supplementary Fig. S1: Multidimensional scaling analysis on sample
Scatterplot of the first two components from a multidimensional scaling analysis (MSA) on self-reported Italian ancestry of COVID-19 patients and the HapMap3 reference panel. The COVID-19 Italian samples cluster pretty well. As expected, they cluster closely but not perfectly overlapping with the CEU and TSI samples from HapMap3.
Outliers in our COVID-19 samples, clearly of different ancestry, are highlighted by the red box.
ASW: African ancestry in Southwest USA; LWK: Luhya in Webuye, Kenya; MKK: Maasai in Kinyawa, Kenya; YRI: Yoruba in Ibadan, Nigeria; CHB: Han Chinese in Beijing, China; CHD: Chinese in Metropolitan Denver, Colorado; JPT: Japanese in Tokyo, Japan; CEU: Utah residents with Northern and Western European ancestry from the CEPH collection; TSI: Toscans in Italy; MEX: Mexican ancestry in Los Angeles, California; GIH: Gujarati Indians in Houston, Texas.

## Slide 2
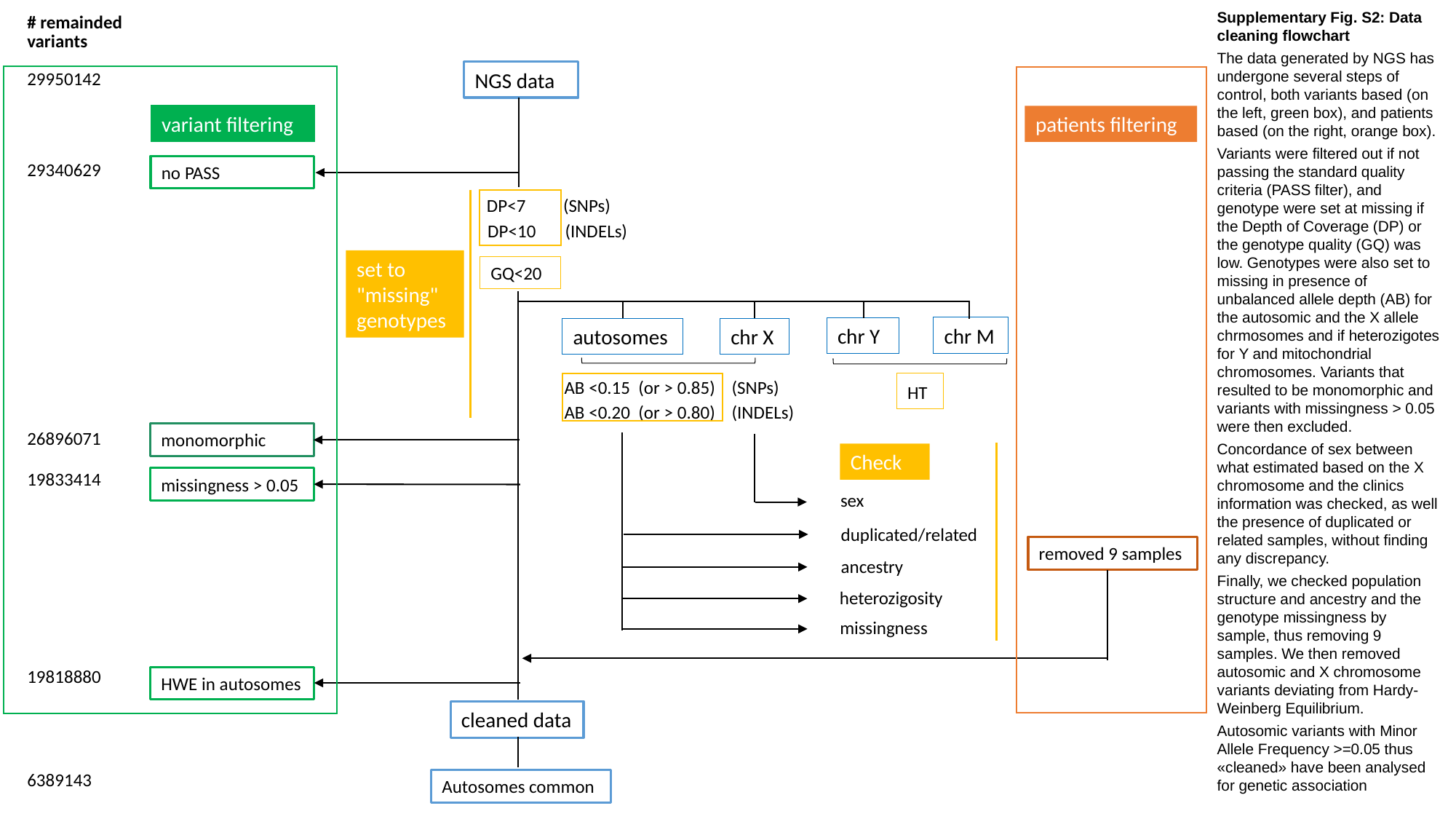

Supplementary Fig. S2: Data cleaning flowchart
The data generated by NGS has undergone several steps of control, both variants based (on the left, green box), and patients based (on the right, orange box).
Variants were filtered out if not passing the standard quality criteria (PASS filter), and genotype were set at missing if the Depth of Coverage (DP) or the genotype quality (GQ) was low. Genotypes were also set to missing in presence of unbalanced allele depth (AB) for the autosomic and the X allele chrmosomes and if heterozigotes for Y and mitochondrial chromosomes. Variants that resulted to be monomorphic and variants with missingness > 0.05 were then excluded.
Concordance of sex between what estimated based on the X chromosome and the clinics information was checked, as well the presence of duplicated or related samples, without finding any discrepancy.
Finally, we checked population structure and ancestry and the genotype missingness by sample, thus removing 9 samples. We then removed autosomic and X chromosome variants deviating from Hardy-Weinberg Equilibrium.
Autosomic variants with Minor Allele Frequency >=0.05 thus «cleaned» have been analysed for genetic association
| # remainded variants |
| --- |
| 29950142 |
| 29340629 |
| 26896071 |
| 19833414 |
| 19818880 |
| 6389143 |
NGS data
variant filtering
patients filtering
no PASS
DP<7 (SNPs)
DP<10 (INDELs)
set to "missing" genotypes
GQ<20
chr M
chr Y
chr X
autosomes
AB <0.15 (or > 0.85) (SNPs)
AB <0.20 (or > 0.80) (INDELs)
HT
monomorphic
Check
missingness > 0.05
 sex
duplicated/related
removed 9 samples
ancestry
heterozigosity
missingness
HWE in autosomes
cleaned data
Autosomes common

## Slide 3
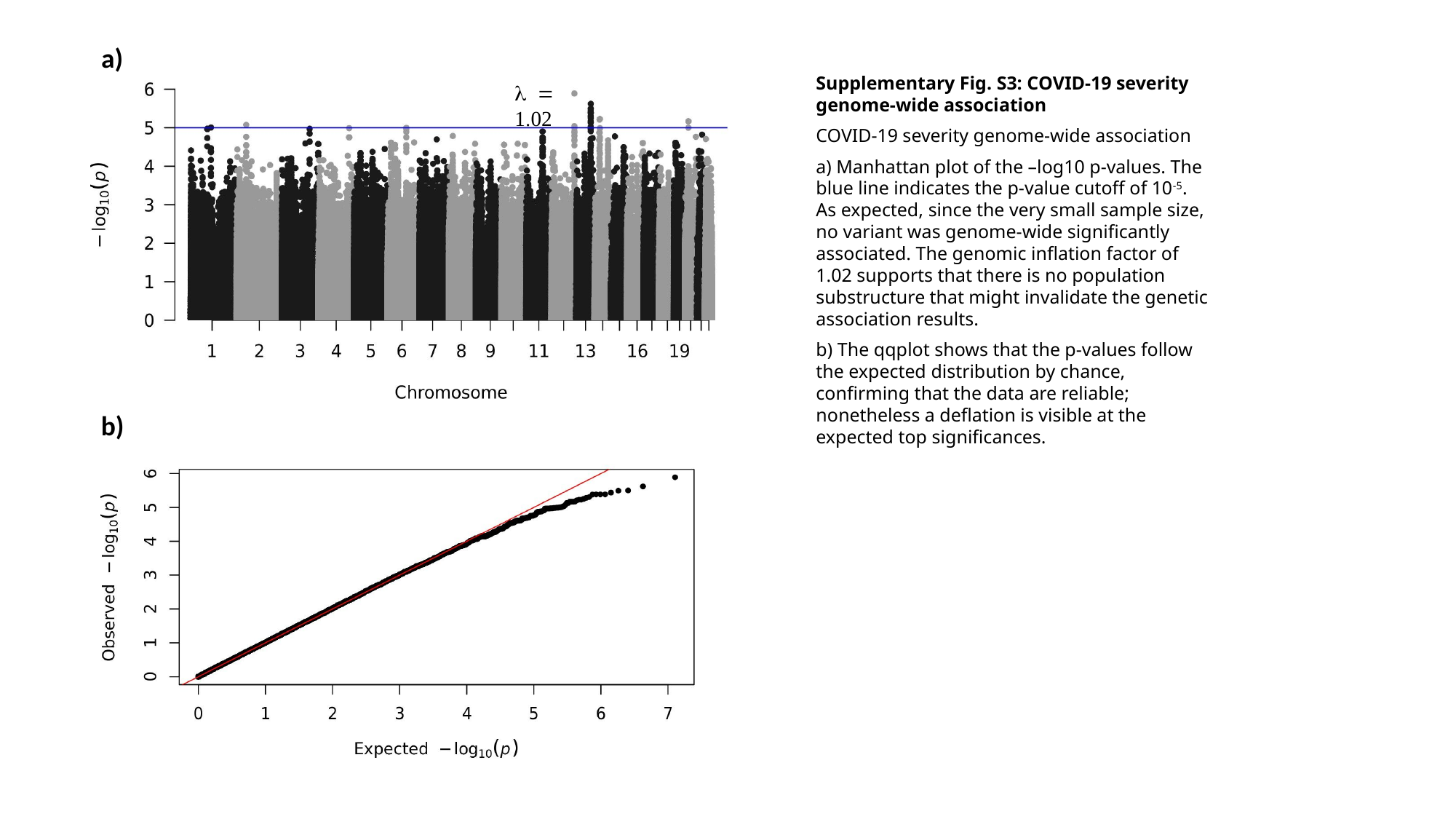

a)
Supplementary Fig. S3: COVID-19 severity genome-wide association
COVID-19 severity genome-wide association
a) Manhattan plot of the –log10 p-values. The blue line indicates the p-value cutoff of 10-5. As expected, since the very small sample size, no variant was genome-wide significantly associated. The genomic inflation factor of 1.02 supports that there is no population substructure that might invalidate the genetic association results.
b) The qqplot shows that the p-values follow the expected distribution by chance, confirming that the data are reliable; nonetheless a deflation is visible at the expected top significances.
l = 1.02
b)

## Slide 4
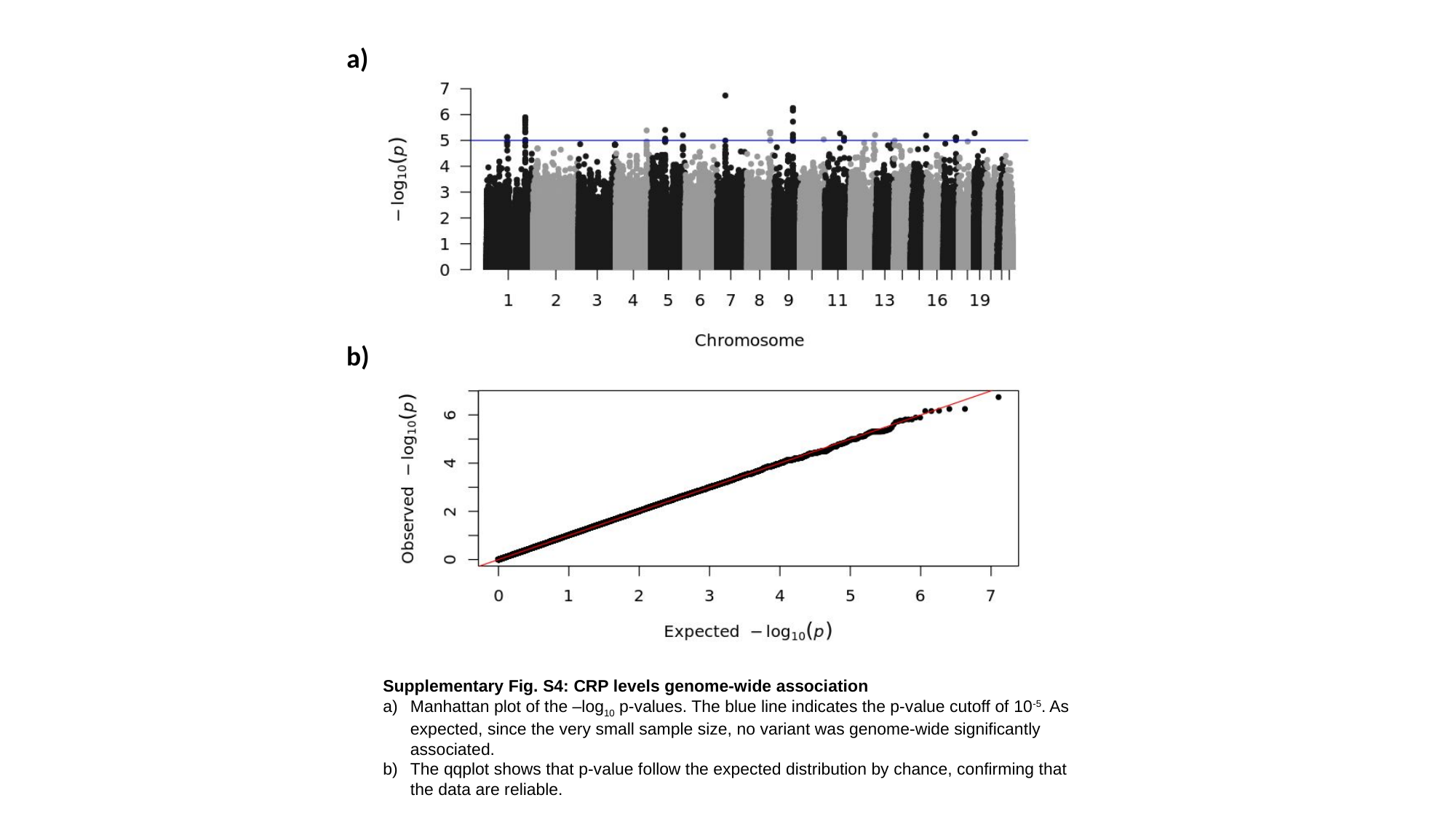

a)
b)
Supplementary Fig. S4: CRP levels genome-wide association
Manhattan plot of the –log10 p-values. The blue line indicates the p-value cutoff of 10-5. As expected, since the very small sample size, no variant was genome-wide significantly associated.
The qqplot shows that p-value follow the expected distribution by chance, confirming that the data are reliable.

## Slide 5
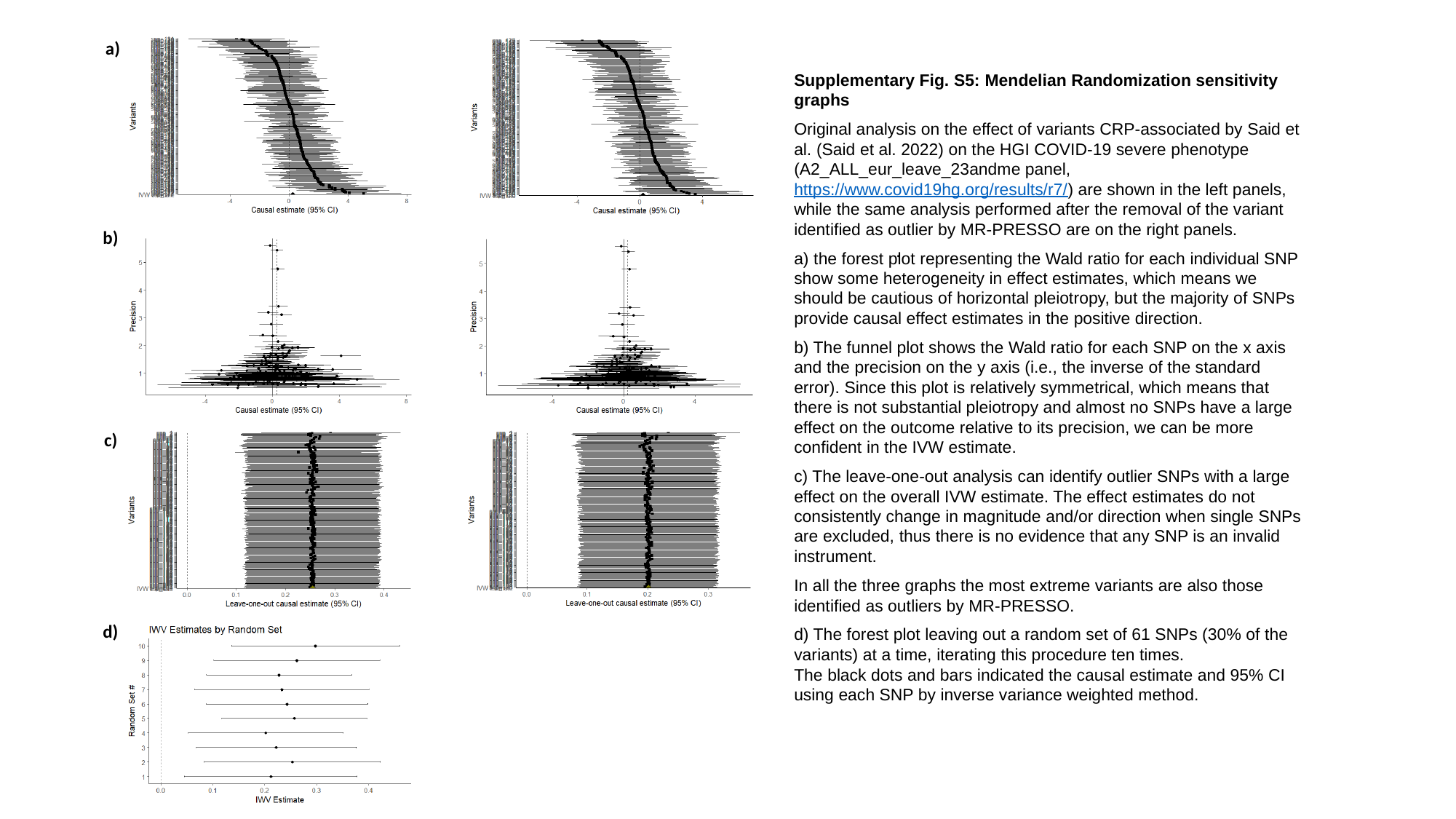

a)
Supplementary Fig. S5: Mendelian Randomization sensitivity graphs
Original analysis on the effect of variants CRP-associated by Said et al. (Said et al. 2022) on the HGI COVID-19 severe phenotype (A2_ALL_eur_leave_23andme panel, https://www.covid19hg.org/results/r7/) are shown in the left panels, while the same analysis performed after the removal of the variant identified as outlier by MR-PRESSO are on the right panels.
a) the forest plot representing the Wald ratio for each individual SNP show some heterogeneity in effect estimates, which means we should be cautious of horizontal pleiotropy, but the majority of SNPs provide causal effect estimates in the positive direction.
b) The funnel plot shows the Wald ratio for each SNP on the x axis and the precision on the y axis (i.e., the inverse of the standard error). Since this plot is relatively symmetrical, which means that there is not substantial pleiotropy and almost no SNPs have a large effect on the outcome relative to its precision, we can be more confident in the IVW estimate.
c) The leave‐one‐out analysis can identify outlier SNPs with a large effect on the overall IVW estimate. The effect estimates do not consistently change in magnitude and/or direction when single SNPs are excluded, thus there is no evidence that any SNP is an invalid instrument.
In all the three graphs the most extreme variants are also those identified as outliers by MR-PRESSO.
d) The forest plot leaving out a random set of 61 SNPs (30% of the variants) at a time, iterating this procedure ten times.
The black dots and bars indicated the causal estimate and 95% CI using each SNP by inverse variance weighted method.
b)
.
c)
d)

## Slide 6
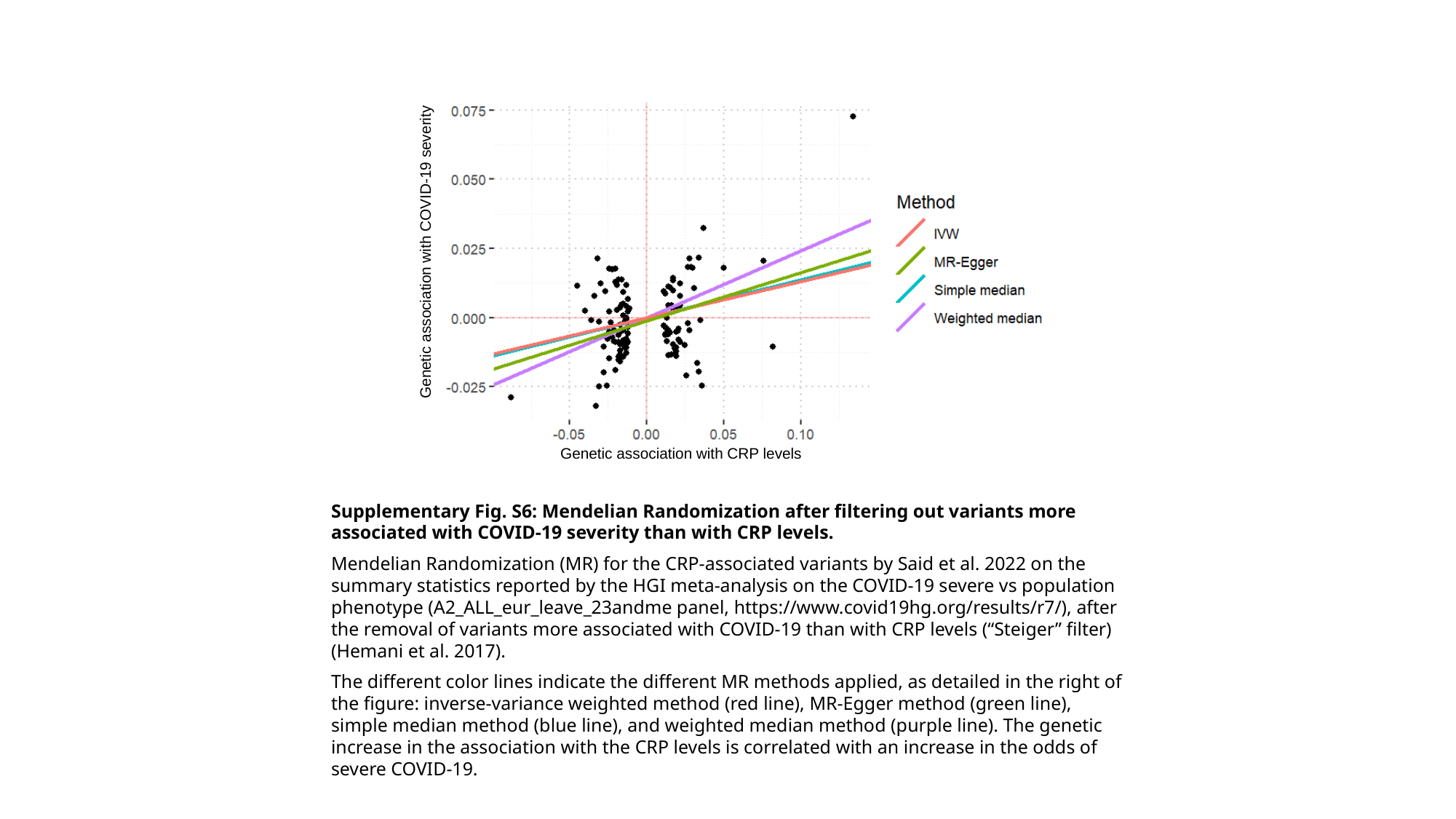

Genetic association with COVID-19 severity
Genetic association with CRP levels
Supplementary Fig. S6: Mendelian Randomization after filtering out variants more associated with COVID-19 severity than with CRP levels.
Mendelian Randomization (MR) for the CRP-associated variants by Said et al. 2022 on the summary statistics reported by the HGI meta-analysis on the COVID-19 severe vs population phenotype (A2_ALL_eur_leave_23andme panel, https://www.covid19hg.org/results/r7/), after the removal of variants more associated with COVID-19 than with CRP levels (“Steiger” filter) (Hemani et al. 2017).
The different color lines indicate the different MR methods applied, as detailed in the right of the figure: inverse-variance weighted method (red line), MR-Egger method (green line), simple median method (blue line), and weighted median method (purple line). The genetic increase in the association with the CRP levels is correlated with an increase in the odds of severe COVID-19.

## Slide 7
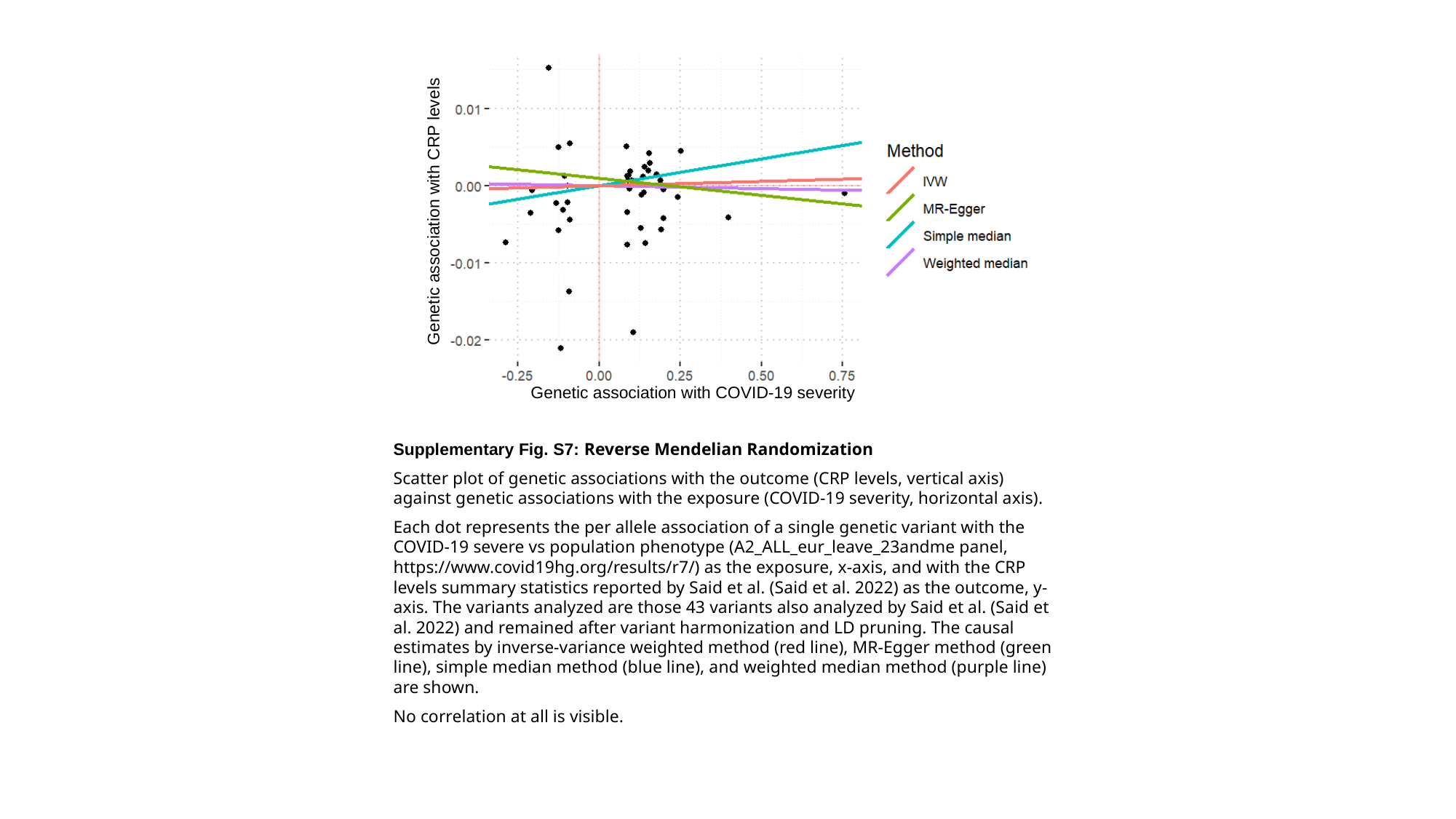

Genetic association with CRP levels
Genetic association with COVID-19 severity
Supplementary Fig. S7: Reverse Mendelian Randomization
Scatter plot of genetic associations with the outcome (CRP levels, vertical axis) against genetic associations with the exposure (COVID-19 severity, horizontal axis).
Each dot represents the per allele association of a single genetic variant with the COVID-19 severe vs population phenotype (A2_ALL_eur_leave_23andme panel, https://www.covid19hg.org/results/r7/) as the exposure, x-axis, and with the CRP levels summary statistics reported by Said et al. (Said et al. 2022) as the outcome, y-axis. The variants analyzed are those 43 variants also analyzed by Said et al. (Said et al. 2022) and remained after variant harmonization and LD pruning. The causal estimates by inverse-variance weighted method (red line), MR-Egger method (green line), simple median method (blue line), and weighted median method (purple line) are shown.
No correlation at all is visible.
