# Supplementary material for: Machine learning, whole genome sequencing, and Mendelian randomization support a role of CRP on COVID-19 severity
Source: Mol Med. 2026 May 27;32:117. doi: 10.1186/s10020-026-01512-6 (PMC13393844; doi:10.1186/s10020-026-01512-6)
Supplement: Supplementary file 2 — Supplementary Material 2. [file 10020_2026_1512_MOESM2_ESM.pdf]

Supplementary Text

Lantieri F. et al. **Machine Learning, Whole Genome Sequencing, and Mendelian Randomization Support a Role of CRP on COVID-19 Severity**

METHODS

Patients' characteristics and inclusion criteria

At the Azienda Unità Sanitaria Locale (AUSL)-IRCCS (Reggio Emilia, Italy), the diagnostic protocol for patients with suspected COVID-19 during the pandemic included nasopharyngeal and oropharyngeal swabs for RT-PCR, blood tests, chest X-rays. In cases of either positive X-ray findings or negative X-rays findings with highly suggestive clinical features, CT scans were also scheduled. A multidisciplinary team defined a classification based on patient clinical features, vital signs, medical history, blood tests and instrumental findings. In particular, six clinical phenotypes were defined (Galli et al. 2022).

To identify genetic variants associated with COVID-19 severity, we enrolled 100 patients with severe disease (phenotypes 4 and 5 based on Galli et al. (Galli et al. 2022) and one with 100 subjects with mild symptoms (phenotypes 1 and 2A). The first group included 100 hospitalized patients consecutively diagnosed with SARS-CoV-2 infection from March 2020 to May 2020 and having received non-invasive ventilation or intubated during the hospitalization. They belonged to phenotypes 4 and 5 based on Galli et al. (Galli et al. 2022). We excluded patients with other infections. A hundred patients who did not require hospitalization and belonged to phenotypes 1 and 2A (Galli et al. 2022) were retrospectively selected so that they were age and sex frequency matched to the severe group. Half of them had an emergency room visit in the same period as the severe group, at which the standard blood tests were performed. Given the enrollment period, none of the patients included in the study was COVID-19 vaccinated.

The mild group was retrospectively selected from consecutive eligible patients by a team of public health operators involved in the mandatory SARS-Cov-2 epidemiological surveillance. They were contacted by phone to propose to participate to the study, offering an appointment, up to December 2020. In that occasion, they received information about the study and eventually signed the consent to participate and gave blood sample for the genetic analysis. Non-responders were substituted with consecutive cases trying to maintain sex and age frequency matching. However, these substitutions resulted in more females and slightly younger mean age in non-severe compared to severe patients. Presence of comorbidities, such as Stroke, Heart Failure, Peripheral Vascular Disease, Cerebrovascular Disease, Dementia, BPCO - Chronic Pulmonary Disease, Peptic Ulcer Disease, Mild Liver Disease, Severe/moderate Liver Disease, Diabetes without complications, Diabetes with complications, Rheumatic Disease, Paraplegia and Hemiplegia, Renal Diseases, Cancer, Metastatic

Carcinoma, AIDS/HIV as well as the Charlson Comorbidity Index, which provides an overall measure of an individual patient's complexity (Charlson et al. 1987) were collected from both patient sets. The index was categorized in four classes: 0 (no presence of relevant comorbidity), 1, 2 and ≥3.

DNA extraction, Genome Sequencing, and data cleaning

DNA was manually extracted from 200 □l of peripheral blood with the QIAamp DNA Blood Mini KIT (QIAGEN). DNA was quantified with Nanodrop system to evaluate DNA purity and concentration as well as with Qubit system to evaluate the concentration of duplex DNA. 800ng of DNA were sent for each patient from the sample recruitment center to the sequencing center for Whole Genome Sequencing (WGS). WGS was performed at the Genomics Facility of the Italian Institute of Technology (IIT, Genoa, Italy).

Approximately 200 ng of purified genomic DNA was processed using the Microlab STAR (Hamilton Company) according to the manufacturer's instructions for the Illumina DNA Prep kit and IDT for Illumina DNA/RNA UD Indexes kit (Illumina, Inc.). The quality and average size of each genomic library were assessed using the Agilent DNA 1000 Kit on an Agilent Bioanalyzer 2100 (Agilent Technologies, Inc.). Approximately 0.85 nM of 24 barcoded genomic libraries were pooled, loaded onto a single NovaSeq 6000 S4 Reagent Kit v1.5 (300 cycles), and sequenced using the Illumina NovaSeq 6000 system (Illumina, Inc.).

Quality check of data obtained was achieved by FastQC tool (Andrews 2010). The FastQ data processing, meaning primary analysis, was achieved through the commercial Illumina DRAGEN (Dynamic Read Analysis for GENomics) Bio-IT Platform, based on GATK Best Practices (Van der Auwera and D 2020). In particular, the Germline Pipeline (v.3.10.4) was applied on all single samples using the genome assembly GRCh38 (hg38) as reference. VCF files were annotated with Variant Effect Predictor (VEP) tool as previously described (Grossi et al. 2023) without any filter on the allele frequency.

Variants detected by genome sequencing were filtered out if not passing the standard quality criteria, i.e. QUAL<10.41 for SNPs and <7.83 for Indels; DP≤1; genotype call from variant caller not consistent with chromosome ploidy; median base quality of alternative allele reads not meeting threshold; variant not meeting likelihood threshold of 6.3. Additionally, to exclude possible false genotype calls, we set at missing genotypes with low Depth of Coverage (DP<7 for SNPs and DP<10 for InDels), low genotype quality (GQ<20), or unbalanced allele depth (AB<0.15 or >0.85 for SNPs and AB<0.20 or >0.80 for InDels) for autosomal and X chromosomes. For Y and mitochondrial chromosomes, also heterozygous genotypes were set as missing. Variants that resulted to be monomorphic and variants with missingness > 0.05 after the initial data cleaning were excluded. Samples sex discordant (chromosome X homogeneity higher than 0.2 for females and lower than 0.8

for males) and duplicated or related between each other based on estimated proportion of identity by descent (IBD higher than 0.185) were planned to be filtered out.

We checked population structure and ancestry by the multidimensional scaling analysis implemented in plink v1.07 (http://pngu.mgh.harvard.edu/purcell/plink/),(Purcell et al. 2007) using autosomic pruned (r2>0.2) SNPs, excluding strand ambiguous variants, and using HapMap3 as the reference (https://www.broadinstitute.org/medical-and-population-genetics/hapmap-3). The first two components were plotted to identify samples with divergent ancestry (supplementary Fig. S1). After this check we removed samples with different ancestry, with genotype missingness higher than 3% or with abnormal heterozygosity (above mean 3±SD). Autosomic variants deviating from Hardy-Weinberg Equilibrium in mild cases (HWE p<10-6) were further removed (supplementary Fig. S2). Data cleaning was performed with bcftools 1.9 and with plink v1.07 (http://pngu.mgh.harvard.edu/purcell/plink/) (Purcell et al. 2007), the PCA plot was performed with R 4.3.2 (R Core Team) (https://www.R-project.org/).

Machine learning analysis

An analysis via machine learning tools was performed on clinical data to discriminate the mild from the severe status via an interpretable classification predictive model. Interpretability is rather important as it avoids getting a powerful predictive, yet obscure model, and hence poor for a clinical scenario. To build an interpretable predictive model, among other possibilities, we took mainly advantage of a Decision Tree Classifier. Decision trees are inherently interpretable models as they discover a set of human interpretable "if-then-else" rules which determine the classification of a sample based on the selected features. An example rule is if(x<5) and (y>10) then 'severe', else 'mild', where x and y are respectively two chosen features among the ones available in the dataset. Like many machine learning methods, decisions trees are capable of both selecting appropriate features and defining rules and the threshold values. As this model can provide complex nested rules, the method is called tree where each node is a rule after which another or more rules can be applied. In our previous example (x<5) was a node of our tree, (y>10) another node, and combined together they provide a leaf. A leaf hence is the combination of rules along a tree to define a class. Also, the same class can be determined by other rules involving other features. To avoid overfitting (non-transferability of the models) we evaluated accuracy, precision and recall only on validation data and allowed a maximum of two levels (tree depth) in the decision tree (no more than 3 variables can be used to build the rules).

To support the analysis, we prepared a clinical data matrix where each entry is a patient, and each variable (feature in the machine learning jargon) is a clinical datum. We included in the model the leukocytes, neutrophils, lymphocytes, and monocytes count and percentages, platelets count, as well as LDH and CRP levels. Further clinical data were available such as Lung parenchyma involvement,

Ground glass, areas of parenchymal consolidation, yet we discarded these data to concentrate on pure quantitative and blood-derived quantities. In total we obtained 143 samples well balanced in terms of sex and the severe/mild status. Additional machine learning results were obtained through a Random Forest (to obtain a well calibrated probability curve) and logistic regression to further support the CRP role.

Mendelian Randomization

Study design

Mendelian Randomization (MR) assesses whether a genetically predicted risk factor, the exposure, has a causal effect on the outcome, investigating whether the exposure-associated genetic variants (used as instrumental variables, IVs) are also associated with the outcome. Since the alleles are randomly distributed among the population, if an exposure is associated with an outcome and alleles known to be associated with the exposure are found to also be associated with the outcome, the exposure can be assumed to have a causal effect on the outcome. We applied a two-sample MR analysis to COVID-19 as the outcome, using the CRP levels as the exposure, under the assumption that: 1) the genetic variants selected as Instrument Variables (IV) are strongly associated with the exposure (CRP levels) (Relevance); 2) the genetic variants are not associated with the outcome (COVID-19) via a confounding pathway (Independence); and 3) the genetic variants do not affect COVID-19 directly, but only via the CRP levels (Exclusion restriction).

Genetic instrument variables

The effect of CRP levels on the severity of COVID-19 was investigated by using the 266 variants reported as genome-wide significantly associated with the level of CRP by Said et al. (Said et al. 2022) The authors performed a one-step meta-analysis on a GWAS performed on UKBiobank participants and the CHARGE consortium GWAS meta-analysis summary statistics. The UKBiobank GWAS was conducted using Linear Mixed Model (LMM) regression with an additive genetic model, adjusting for age, sex, UKBiobank array and 40 genetic principal components. The serum CRP levels (mg/l, measured by immunoturbidimetry) of 575531 persons of European descent in UKBiobank were transformed using the natural log. The CHARGE consortium GWAS meta-analysis was conducted “1000 Genomes” imputed data from 49 studies. The 266 variants are listed in Supplementary Table S5. Given the very big sample size of the study of around 500,000 individuals and the genome-wide significant association, with F-statistics well higher than 10, we considered these variants as relevantly associated (assumption #1). However, R^2, and the F-statistics for each variant were estimated to check for instrument strength. The proportion of variance in exposure explained by genetic variants, R^2, was

calculated as $2\hat{\alpha}^2 \times MAF \times (1 - MAF)$, where MAF_j is the minor allele frequency and $\hat{\alpha}$ is the SNP-exposure association estimate. The F-statistics for each variant has been calculated as $F = \frac{N-K-1}{K}\frac{R^2}{1-R^2}$, where N is the sample size and K is the number of IVs (Burgess et al. 2016). An approximation of the first-stage F statistic for all variants based on the summarized data was estimated by the Mendelian Randomization package, version 0.10.0 (Yavorska and Burgess 2017), R Software 4.3.2. (https://www.R-project.org/) (R Core Team). We did not carry out any LD-based pruning in the main analysis because these variants are reported as independently associated with the CRP levels. However, we performed a sensitivity analysis on only the SNPs pruned by linkage disequilibrium (LD) with r2<0.01 over 500kb. We used the online SNP clip tool (https://ldlink.nih.gov/?tab=snpclip) available on the LDlink suite (Machiela and Chanock 2015), searching for the variants position reported by Said et al.(Said et al. 2022) (genome build GRCh37), using as the population reference the 5 European populations (EUR) from the 1000genomes project, which are CEU (Utah Residents from North and West Europe), TSI (Tuscany in Italy), FIN (Finnish in Finland), GBR (British in England and Scotland), and IBS (Iberian population in Spain), and setting MAF at 0.05. We have thus removed 5 SNPs: one (rs34633805) because it could not be checked since not found in dbSNP155 (GRCh37), the other four because in LD with other variants among the 266 by Said et al. (Said et al. 2022): rs7933202 in LD with rs4647725 (r^2=0.0103), rs1056441 in LD with rs6073958 (r^2=0.011), rs112957492 in LD with rs4658403 (r^2=0.0105), and rs459625 in LD with rs7756870 (r^2=0.0118).

Given that the instrumental variables should act on the outcome only through the exposure, we repeated the analysis excluding those variants with stronger associations with the outcomes than with the exposure (Steiger filtering) (Hemani et al. 2017). Additionally, we searched the PheWeb version 1.3.15 (https://pheweb.sph.umich.edu/), using the UKBiobank TOPMed-imputed dataset to check whether the variants might have a confounding effect on the outcome through different risk factors, and repeated the MR analysis excluding those variants associated with a $p<10^{-5}$ for endocrine/metabolic disorders such as obesity, diabetes, circulatory system or digestive traits.

Finally, given that none of the IVs above described were located in the CRP gene and are therefore to be considered as trans-IVs, we also performed the MR analysis using cis-acting IVs. To this end we selected the variants located in the CRP gene +/- 50 kb, and applied the same method described above.

Data source and phenotype.

We extracted the summary statistics for these 267 variants from the meta-analysis reported by the Host Genetic Initiative (HGI) GWAS meta-analyses round 7 (https://www.covid19hg.org/results/r7/), Release Date: April 8, 2022. Since our hypothesis of a role of the CRP levels in COVID-19 severity was motivated by the findings on our Italian sample, we were particularly interested in the European population, and we thus selected the A2_ALL_eur_leave_23andme panel

(https://www.covid19hg.org/results/r7/). This dataset regarded 13769 total cases and 1072442 controls for the phenotype “Very severe respiratory confirmed covid vs. population”, as detailed on the HGI website (https://docs.google.com/document/d/1okamrqYmJfa35ClLvCt_vEe4PkvrTwggHq7T3jbeyCI/edit).

We also extracted the data for the phenotype “Hospitalized covid vs. not hospitalized covid” reported in the dataset B1_ALL_leave_23andme, which included 16512 cases and 71321 controls without distinguishing by ancestry (https://www.covid19hg.org/results/r7/). In addition, we checked the NHGRI-EBI GWAS Catalog (https://www.ebi.ac.uk/gwas/) (Sollis et al. 2023) on 30/11/2023 to search for additional GWAS, prioritizing by the sample size analyzed. The biggest sample size GWAS identified were all previous releases by the HGI and thus already included in the meta-analysis association from HGI release #7. However, we also selected the study by Ellinghaus et al.(Ellinghaus et al. 2020) (Accession ID GCST90000255), although much smaller in sample size (835 cases and 1255 controls), because specifically carried out on severe COVID-19 (defined as respiratory failure) patients vs blood donors controls from Italian and Spanish hospitals.

Finally, an overlap between HGI samples and UK Biobank does exist. This overlap, calculated on the biggest HGI sample, was 38.7% (4.9% in cases and 39.1% in controls) for the A2 panel, and 16.2% (24.6% in cases and 14.3% in controls) for the B1 panel. The overlap between the samples in which the associations of IVs with the risk factor and with the outcome are calculated is unlikely to give strong bias in a two sample MR in presence of strong genetic instrumental variants. However, we have estimated the type I error rate through the app available by Burgess et al. (Burgess et al. 2016). No sample overlap is apparent for the sample by Ellinghauss et al. (Ellinghaus et al. 2020).

Mendelian randomization statistical analysis

The multiplicative random-effect inverse-variance weighted (IVW) method was used as the primary analysis. This method is a meta-analysis of the ratio estimates from each individual variant and assumes that all instruments are valid, without horizontal pleiotropy (Bowden et al. 2016). We employed the weighted median (WM), the simple median (SM), and the MR-Egger method (Bowden et al. 2015) as sensitivity analyses to check for the robustness of results and to investigate directional pleiotropy.

The MR-Egger method calculates the intercept as an estimate of the average pleiotropic effect of the variants on the outcome, under the assumption that the pleiotropic effects are independent of the variant–exposure associations (Instrument Strength Independent of Direct Effect assumption, InSIDE) (Bowden et al. 2015). However, this method has generally lower power to detect a causal effect. The median-based methods are less affected by the presence of pleiotropic variants than IVW and MR-

Egger, with up to half of the variants that can be invalid instruments (majority valid assumption instruments) (Bowden et al. 2016).

The heterogeneity was assessed through Cochran's Q test and I^2 statistics. Heterogeneity should not be different from what expected by chance if all variants were valid instruments and with a linear and homogeneous relationship with outcome and exposure for all individuals in the population. Heterogeneity may thus occur if there is no linearity and homogeneity across individuals, if variants influence the exposure through different biological mechanisms, but also in case of invalid instruments. We also applied the Mendelian Randomization Pleiotropy Residual Sum and Outlier (MR-PRESSO) using the MRPRESSO R package (Verbanck et al. 2018) in R Software 4.3.2. (https://www.R-project.org/) (R Core Team) to identify and correct for pleiotropic outliers. The MR-PRESSO method detects variants whose effect estimates differ substantially from those of other variants and recalculate the estimates after their removal; the global test evaluates whether horizontal pleiotropy is present between all instruments (Verbanck et al. 2018). We estimated the heterogeneity again after removing genetic variants that display horizontal pleiotropy identified by MR-PRESSO. Funnel plots and forest plots were used to explore the symmetry and the heterogeneity of estimates of the causal effects of individual variants. We also performed Leave-one-out analysis, which systematically reanalyzes the data removing one genetic variant at a time to check whether the results were overly driven by specific variant/s (Corbin et al. 2016). Since we were analyzing more than two hundred variants, and a single variant was unlikely to impact so much on the final estimate, we also choose to leave out a random set of 61 SNPs (30% of the variants) at a time, iterating this procedure ten times. All analyses were two-sided. Odds ratio (OR) and 95% CI are calculated as the exponent of beta and reported as the effect estimates for MR on COVID-19, together with beta estimates.

Bidirectional analysis

To perform a two-sample MR using severe COVID-19 as the exposure and the pCRP levels as the outcome, we selected the variants that were genome-wide significantly associated with COVID-19 severity ($p<5\ 10^{-8}$) from the A2_ALL_eur_leave_23andme Host Genetic Initiative (HGI) GWAS meta-analyses release #7 (https://storage.googleapis.com/covid19-hg-public/freeze_7/results/20220403/pop_spec/sumstats/COVID19_HGI_A2_ALL_eur_leave23andme_20220403.tsv.gz). Of the 3932 variants thus selected, 3317 were also present in the summary statistics from the paper by Said et al. (Said et al. 2022), as retrieved from the NHGRI-EBI GWAS Catalog (accession ID GCST90029070), after removal of palindromic variants. These variants were pruned by linkage disequilibrium (LD) through the online SNP clip tool (https://ldlink.nih.gov/?tab=snpclip) available on the LDlink suite (Machiela and Chanock 2015), searching for the variants position in the GRCh38 genome build, using as the population reference the 5 European population (EUR) from the

1000genomes project and setting MAF at 0.05. Forty-three independent variants (r^2<0.01 at a window size of 500kb) were retained, harmonized, and used for the two sample Mendelian Randomization analysis.

RESULTS

The genetic profile of the samples confirms the robustness and accuracy of the phenotype ascertainment

The whole genome sequencing was performed on all the 200 samples. The overall mean coverage was 30.1X (min 17.5X, max 40.0X) with a mean coverage uniformity (percentage of sites with coverage greater than 20% of the mean coverage in region) of 96.8% (min 96.3%, max 97.1%) and an overall percentage of sites with at least 20X coverage of 88.4% (see details in the Supplementary Table S1)

Accurate data cleaning allowed us to exclude nine patients because of different ancestry from the rest of the samples, abnormal heterozygosity, and/or genotype call missingness above 3%.

Seven hundreds and ninety-one, 53.676, 792, and 19.778.154 variants were retrieved for chromosome Y, chromosome X, mitochondrial chromosomes, and autosomes respectively (Supplementary Fig. S1 and S2).

Following data cleaning, 6.389.143 variants located on autosomal chromosomes, with a call rate ≥97%, MAF≥5%, and not deviating from HWE in the mild cases ($p \geq 10^{-6}$) were analyzed for genetic association with severe COVID-19, comparing hospitalized patients with severe COVID-19 (95 cases) vs mild, not hospitalized COVID-19 patients regarded as controls (96 patients). The genetic association with the iCRP levels was carried out on 136 patients for which the iCRP level had been measured at the emergency unit (93 COVID-19 severe and 43 COVID-19 mild patients).

Since neither sex and age, as expected by study design, nor complications resulted to be significant covariates on the COVID-19 severity phenotype, we performed case-control genetic association on COVID-19 severity by chi-square test. Genetic associations on iCRP levels inverse normal transformed were carried out by linear regression adjusting for sex and age as covariates. We repeated the association analyses including sex, age, and comorbidities (defined as the presence of any comorbidity among those collected) as well as the first 10 principal components, estimated by the multidimensional scaling analysis as a sensitivity analysis.

A genome-wide analysis was carried out with the purpose of checking for the p-values distribution and for the Lambda Genomic control inflation factor □ (based on median chi-squared values divided by the expected median of the chi-squared distribution). The GWAS for COVID-19 severity (hospitalized/severe vs not hospitalized/mild) showed a genomic inflation factor lambda (□) =1.02, supporting the good quality of the sample, without apparent population stratification. The distribution

of p-values followed the expected normal distribution, nonetheless, they were deflated at the top significance, showing less significant p-values than expected (Supplementary Fig. S3 and Supplementary Table S2). This deflation was not evident for the association with the iCRP levels (Supplementary Fig. S4 and Supplementary Table S2). None of the variants reported as associated with any COVID-19 phenotype by HGI was significant in our sample after correction for multiple tests. However, for the B1 phenotype, an enrichment in nominally significant candidate variants with respect to the variants associated with the other COVID-19 phenotypes was observed (□^{2} with Yates' correction =48.651, df=1, $p<3.1\cdot10^{-12}$). The B1 HGI phenotype (hospitalized vs not hospitalized) is the one that most corresponds to our sample recruitment definition of severe hospitalized and mild not hospitalized. We also analyzed the twelve variants used by Roberts et al. (Roberts et al. 2022) as candidate proxy for 8 COVID-19 phenotypes defined based on a self-reported questionnaire. In line with the authors, we were able to replicate one of the two variants (rs35081325, locus SLC6A20/LZTFL1) associated with hospitalized vs not hospitalized patients, which is also strongly associated with the severity score phenotype, confirming the robustness of our data and the fact that our phenotype is consistent with the hospitalized/severe vs not hospitalized/mild phenotype. The frequency of the effect allele T at the above SLC6A20/LZTFL1 locus variant was 18.4% in severe COVID-19 patients, 10.9% in mild patients (OR [95%CI]=1.84 [1.03-3.30], p=0.039), and 7.5% in gnomAD non-Finnish Europeans.

Genetic overlap between COVID-19 and CRP levels.

We investigated the variants associated with COVID-19 severity and CRP levels at genome-wide level. More variants than expected were nominally significant with both iCRP levels and COVID-19 (54729 variants, McNemar's chi-squared =6.73, df=1, p=0.009478), and in almost complete accordance with the effect direction (54568 out of 54729 variants, 99.7%) (Supplementary table S2). These results were consistent pruning variants based on LD ($r^2>0.1$), with a proportion of variant associated with both iCRP levels and COVID-19 higher than expected (1632 out of 194846, McNemar's chi-squared = 6.973, df = 1, p-value = 0.008275), and in almost complete accordance with the effect direction (1626 out of 1632 variants, 99.6%).

Very similar results were obtained by adding sex, age, comorbidities, and the first 10 components as covariates, with 53447 variants in common, 1644 after LD pruning, and similar proportion also at other p-values threshold (for instance, 2 variants in common at $p \leq 5x10^{-4}$ both at the main analysis and adding all the covariates, and 42 and 45 respectively at $p \leq 5x10^{-3}$).

The 43 variants associated with both phenotypes with p<0.0005 were clustered into 12 loci (Supplementary Table S3). These regions regarded the genes SLC6A17, POM121, KIAA1549, GFRA2, GMFG, SLCO4A1, and the intergenic regions between MEIS1 and ETAA1, between

MYO10 and BASP1, between ELOVL5 and GCLC, between POT1 and GRM8, and between ZNF626 and ZNF66. Interestingly, several of these genes have already been implicated in COVID-19 (Satu et al. 2021; Nwabufo et al. 2024). The addition of the above covariates yielded to 45 variants, with 7 loci lost (POM121, KIAA1549, GMFG, DGCR2, and the region between MYO10 and BASP1, between POT1 and GRM8, and between ZNF626 and ZNF66) and 9 gained (PLCH2, LINC01250, FER1L6-AS2, PDE3A, and between GADD45A and GNG12, MARCHF1 and SMIM31, AGA and TENM3, DEUP and SMCO4, and between AOC1 and KCNH2). These differences were all due to marginal shifts around the significance threshold rather than to drastic changes in p-values.

Heterogeneity and pleiotropy detected by Mendelian Randomization

MR-PRESSO global test indicated the presence of horizontal pleiotropy (global test p-value$<10^{-4}$), with three variants identified as outliers, in addition to other five that were suggestive of being outliers, although not at a significant level (Supplementary Tables S5 and S6). However, MR-PRESSO results remained significant after correcting for these outliers (Supplementary Table S4). A high heterogeneity was detected (Cochran's Q p=0.0000 and I^2=41.5%). Of note, after the removal of the three outlier variants, the heterogeneity decreased to I^2=28% (Cochran's Q p=0.0002), and further dropped to 14% (Cochran's Q p=0.0592) when removing all the 8 variants deviating in MR-PRESSO.

The forest plot showed some heterogeneity in effect estimates, indicating possible horizontal pleiotropy, however the majority of SNPs provided causal effect estimates in the positive direction. Accordingly, the leave-one-out analysis showed that effect estimates did not consistently change in magnitude and/or direction when single SNPs are excluded, thus there is no evidence that the SNP are invalid instruments. The same conclusion could be drawn by excluding 30% of the variant at a time. Finally, no substantial pleiotropy and almost no SNPs with a large effect on the outcome relative to its precision was highlighted by the funnel plot, which was relatively symmetrical. In all the three graphs the most extreme variants were also those identified as outliers by MR-PRESSO.

Sensitivity analyses carried out on the 199 trans-IVs gave very similar results (OR [95%CI]: 1.29 [1.02, 1.63], p≤0.0001, I^2 = 40.8% on the 199 variants, and OR [95%CI]: 1.14 [1.10, 1.18], p = 0.024, I^2 = 0.0% on the 124 variants after Steiger Filtering); results were coherent also adding the 5 cis-IVs (Supplementary Table S4).

In conclusion, both the MR-PRESSO analysis, the heterogeneity estimates, and the three graphs, as well as the sensitivity analyses, were consistent with the main MR results and confirmed its reliability and robustness.

Evaluation of sample overlap

For the MR, a sample overlap between the samples used for the association on pCRP and for the COVID-19 severity does exist. We did not perform sensitivity analysis excluding the overlapping samples, thus we cannot exclude bias due to this partial sample overlap in our MR analysis. However, this chance is negligible: the IVs are pretty strong (ie high F-statistics>10) and the overlap with the A2 panel was mainly due to the controls, with only 4.9% of cases overlapping with the UKBiobank. As a matter of fact, it has been suggested that MR with a binary outcome in which the associations of the IV with the risk factor are only estimated in control participants provides a natural robustness against weak instrument bias.(Burgess et al. 2016) In addition, the bCRP had been measured in the general population of UKBiobank before the spread of COVID-19. Also, the MR analysis on Ellinghauss et al. (Ellinghaus et al. 2020) summary statistics might be seen as a sensitivity analysis confirming our observation made on the HGI sample.

Finally, we have estimated that Type 1 error rates are at nominal levels, further excluding the presence of relevant bias due to the partial overlap existing between the HGI and the UKBiobank samples.

Additional machine learning results

Here we show that using a less interpretable model, namely a Random Forest, we can obtain an almost perfect probability calibration curve, yet we lose interpretability. Below the averaged (1000 trials) calibration curve.

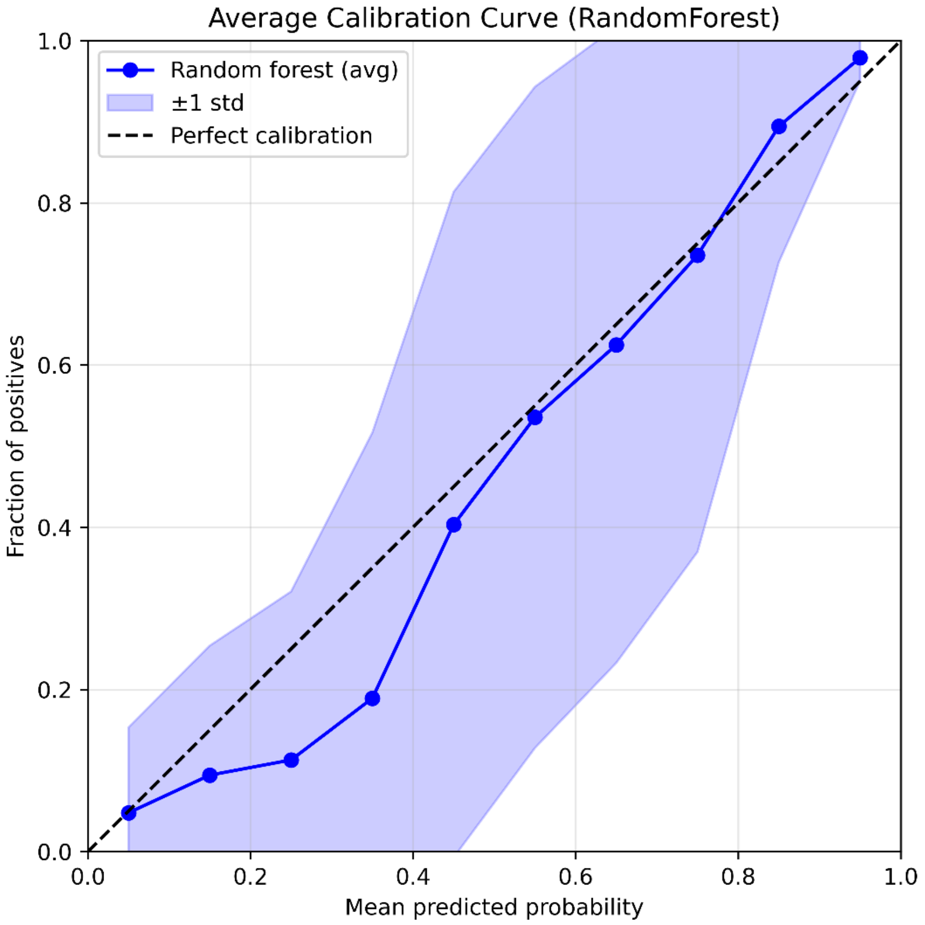

The calibrated curve shows that a very smooth probability calibration can be obtained (at variance of the tree model) yet threshold-level interpretability is lost.

To further check and increase the confidence on our tree-related results we explicitly estimated the full distribution (see below).

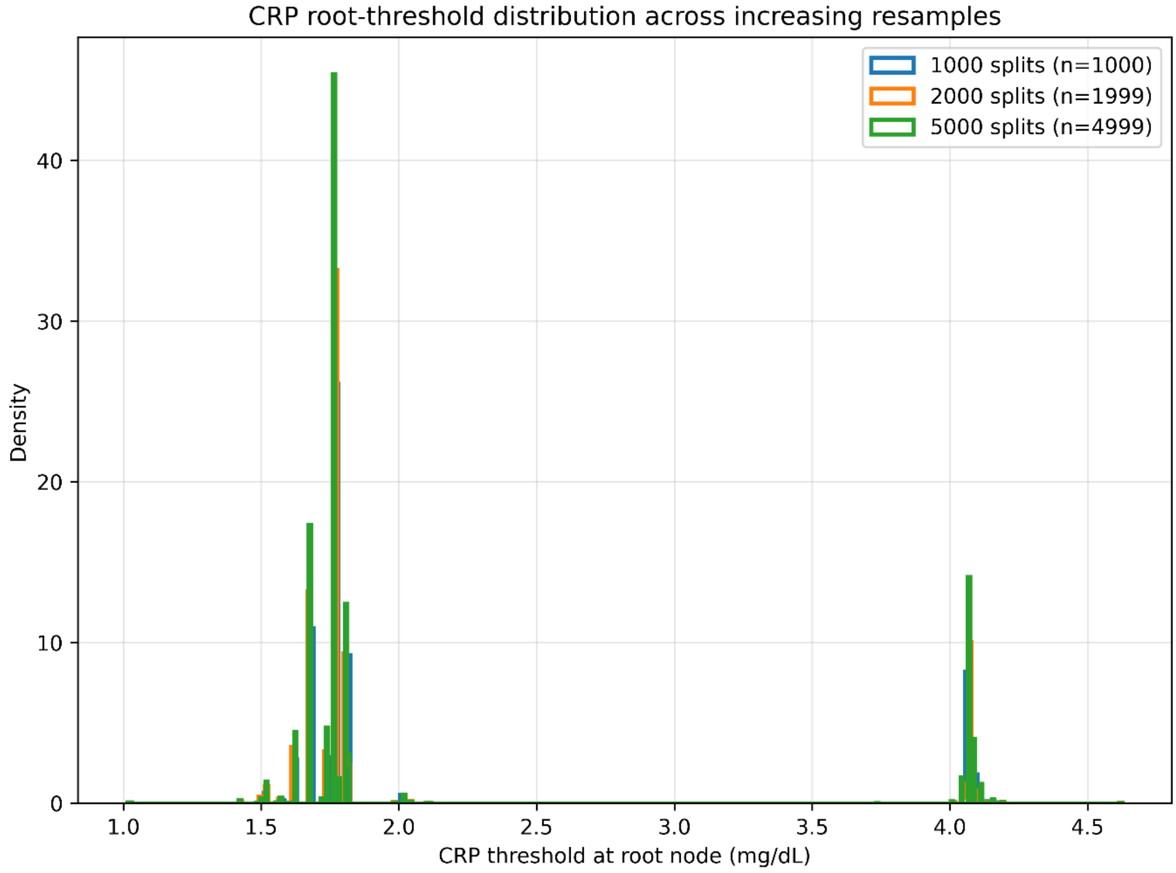

As the plot shows, after 1000 splits the distribution is substantially convergent, and additionally the distribution is bi-modal, where the lower threshold (around 1.7 mg/dL) is more probable than the higher probability counterpart (at around 4.1 mg/dL). This largely explains the discrepancy between mean and median. To robustly estimate a single global mean we used 5000 repetitions and the Chebyshev inequality (it does not assume normality) obtaining [2.126, 2.242] as confidence interval with 95% probability and an average refined value of 2.18 mg/dL.

Lastly, to independently confirm with a non-tree-based tool the role of CRP we run stratified logistic regression. To evaluate the significance of the selected variables, upon z-score normalization of data, we got the absolute values of weights of the learnt models across repetitions and used those values to compute an average ranking value of the features (see table below). CRP confirms itself to be consistently always the most relevant variable (almost always first variable) whereas LDH and Neutrophils are also relevant; all these findings are fully consistent with the variables identified by the decision trees.

Feature	Mean rank
CRP (mg/dL)	1.0210
LDH (U/L)	2.3024
Neutrophyls (%)	3.7416
Lynphocytes (1000/mmc)	3.9612
Monocytes (1000/mmc)	6.2912
Platelets (1000/uL)	7.1218
Lynphocytes (%)	7.1310
Monocytes (%)	7.1920
Leukocytes (1000/uL)	7.4702
Neutrophyls (1000/mmc)	8.7676

REFERENCES

Andrews S. FastQC: a quality control tool for high throughput sequence data. 2010; Available online at: http://www.bioinformatics.babraham.ac.uk/projects/fastqc

Bowden J, Davey Smith G, Burgess S. Mendelian randomization with invalid instruments: effect estimation and bias detection through Egger regression. Int J Epidemiol. 20150606th ed. 2015 Apr;44(2):512–25.

Bowden J, Davey Smith G, Haycock PC, Burgess S. Consistent Estimation in Mendelian Randomization with Some Invalid Instruments Using a Weighted Median Estimator. Genet Epidemiol. 20160407th ed. 2016 May;40(4):304–14.

Burgess S, Davies NM, Thompson SG. Bias due to participant overlap in two-sample Mendelian randomization. Genet Epidemiol. 20160914th ed. 2016 Nov;40(7):597–608.

Charlson ME, Pompei P, Ales KL, MacKenzie CR. A new method of classifying prognostic comorbidity in longitudinal studies: development and validation. J Chronic Dis. 1987;40(5):373–83.

Corbin LJ, Richmond RC, Wade KH, Burgess S, Bowden J, Smith GD, et al. BMI as a Modifiable Risk Factor for Type 2 Diabetes: Refining and Understanding Causal Estimates Using Mendelian Randomization. Diabetes. 20160708th ed. 2016 Oct;65(10):3002–7.

Ellinghaus D, Degenhardt F, Bujanda L, Buti M, Albillos A, Invernizzi P, et al. Genomewide Association Study of Severe Covid-19 with Respiratory Failure. N Engl J Med. 20200617th ed. 2020 Oct 15;383(16):1522–34.

Galli MG, Djuric O, Besutti G, Ottone M, Amidei L, Bitton L, et al. Clinical and imaging characteristics of patients with COVID-19 predicting hospital readmission after emergency department discharge: a single-centre cohort study in Italy. BMJ Open. 20220406th ed. 2022 Apr;12(4):e052665.

Grossi A, Rusmini M, Cusano R, Massidda M, Santamaria G, Napoli F, et al. Whole genome sequencing in ROHHAD trios proved inconclusive: what's beyond? Front Genet. 20230807th ed. 2023;14:1031074.

Hemani G, Tilling K, Davey Smith G. Orienting the causal relationship between imprecisely measured traits using GWAS summary data. PLoS Genet. 20171117th ed. 2017 Nov;13(11):e1007081.

Machiela MJ, Chanock SJ. LDlink: a web-based application for exploring population-specific haplotype structure and linking correlated alleles of possible functional variants. Bioinformatics. 20150702nd ed. 2015 Nov;31(21):3555–7.

Nwabufo CK, Luc J, McGeer A, Hirota JA, Mubareka S, Doxey AC, et al. COVID-19 severity gradient differentially dysregulates clinically relevant drug processing genes in nasopharyngeal swab samples. Br J Clin Pharmacol. 20240531st ed. 2024 Sep;90(9):2137–58.

Purcell S, Neale B, Todd-Brown K, Thomas L, Ferreira MA, Bender D, et al. PLINK: a tool set for whole-genome association and population-based linkage analyses. Am J Hum Genet. 20070725th ed. 2007 Sep;81(3):559–75.

R Core Team. R: A language and environment for statistical computing.

Roberts GHL, Partha R, Rhead B, Knight SC, Park DS, Coignet MV, et al. Expanded COVID-19 phenotype definitions reveal distinct patterns of genetic association and protective effects. Nat Genet. 20220411th ed. 2022 Apr;54(4):374–81.

Said S, Pazoki R, Karhunen V, Võsa U, Ligthart S, Bodinier B, et al. Genetic analysis of over half a million people characterises C-reactive protein loci. Nat Commun. 20220422nd ed. 2022 Apr 22;13(1):2198.

Satu MS, Khan MI, Rahman MR, Howlader KC, Roy S, Roy SS, et al. Diseasome and comorbidities complexities of SARS-CoV-2 infection with common malignant diseases. Brief Bioinform. 2021 Mar 22;22(2):1415–29.

Sollis E, Mosaku A, Abid A, Buniello A, Cerezo M, Gil L, et al. The NHGRI-EBI GWAS Catalog: knowledgebase and deposition resource. Nucleic Acids Res. 2023 Jan;51(D1):D977–85.

Van der Auwera GA, D OB. Genomics in the Cloud: Using Docker, GATK, and WDL in Terra. 1st Edition. 2020;

Verbanck M, Chen CY, Neale B, Do R. Detection of widespread horizontal pleiotropy in causal relationships inferred from Mendelian randomization between complex traits and diseases. Nat Genet. 20180423rd ed. 2018 May;50(5):693–8.

Yavorska OO, Burgess S. MendelianRandomization: an R package for performing Mendelian randomization analyses using summarized data. Int J Epidemiol. 2017 Dec;46(6):1734–9.
